# Pak1 pathway hyper-activation mediates resistance to endocrine therapy and CDK4/6 inhibitors in ER+ breast cancer

**DOI:** 10.1038/s41523-023-00556-9

**Published:** 2023-05-31

**Authors:** Stefania Belli, Daniela Esposito, Alessandra Allotta, Alberto Servetto, Paola Ciciola, Ada Pesapane, Claudia M. Ascione, Fabiana Napolitano, Concetta Di Mauro, Elena Vigliar, Antonino Iaccarino, Carmine De Angelis, Roberto Bianco, Luigi Formisano

**Affiliations:** 1 Department of Clinical Medicine and Surgery, University of Naples “Federico II”, 80131 Naples, Italy; 2 Department of Public Health, University of Naples Federico II, Naples, Italy

**Keywords:** Breast cancer, Cell signalling

## Abstract

Cyclin-dependent kinase 4 and 6 inhibitors (CDK4/6i) have been approved in combination with endocrine therapy (ET) to treat estrogen receptor-positive (ER+) metastatic breast cancer (BC). However, drug resistance represents the leading cause of breast cancer patients mortality. This study aimed to identify novel resistance mechanisms to ER antagonists in combination with CDK4/6 inhibitors. We generated two ER+ BC cell lines, T47D and MCF7, resistant to the combination of the ER antagonist fulvestrant and CDK4/6i abemaciclib, named T47D-FAR and MCF7-FAR. Transcriptomic analysis revealed common up-regulation of genes involved in MAPK and epithelial to mesenchymal transition (EMT) pathways in FAR cells, sustaining their hyper-invasive phenotype and increased anchorage-independent growth, compared to sensitive cells. FAR cells showed higher p21-activated kinase 1 (Pak1) expression and phosphorylation levels than parental cells. *PAK1* knockdown by siRNAs hampered cell proliferation, reduced anchorage-independent growth and invasive properties of T47D-FAR and MCF7-FAR, re-sensitizing them to fulvestrant and abemaciclib. Conversely, over-expression of *PAK1* in MCF7 and T47D cells increased tumor spheroids’ growth and invasion and reduced sensitivity to fulvestrant and abemaciclib, confirming its role in inducing drug resistance. Finally, treatment with Pak1 inhibitors, PF-3758309 (PF309) and NVS-PAK1-1, restored cell sensitivity to fulvestrant and abemaciclib of MCF7-FAR and T47D-FAR cells, both in vitro and in vivo. In conclusion, our data suggested a pivotal role for Pak1 in resistance to ET and CDK4/6i in ER+ breast cancers. These data might promote the rationale for the development of novel Pak1 inhibitors for treatment of patients with ER+ BC progressing on ET plus CDK4/6i.

## Introduction

Approximately 70% of breast cancers express estrogen receptor α (ER+). The combination of cyclin-dependent kinase 4 and 6 (CDK4 and CDK6) inhibitors (CDK4/6i) –palbociclib, ribociclib, or abemaciclib - *plus* endocrine therapy (ET) represents the first-line treatment for patients with ER+/HER2- metastatic breast cancer, which improved the progression-free survival (PFS) and overall survival (OS) compared to ET alone^1^. Despite positive clinical outcomes, the efficacy of these treatments is neutralized by the onset of drug resistance. Hence, understanding mechanisms underpinning resistance to CDK4/6i represents a crucial scientific and clinical need. P21-activated kinase 1 (*PAK1*) has been described as essential for cell survival and transformation. Previous studies reported that *PAK1* gene is amplified in 30% of breast cancers^2–4^. Pak1 belongs to a family of 6 serine/threonine kinases, downstream effectors of Ras-related Rho GTPases Cdc42 and Rac^5^. It is involved in cell viability, cytoskeletal structure, cell adhesion, motility, and mitosis^6^. As a kinase, Pak1 enhances RAF/MEK/ERK signaling pathway, ultimately promoting cell proliferation^7^. In addition, Pak1-mediated phosphorylation of MEK1 is crucial also for cell adhesion processes depending on focal adhesion kinase (Fak) and steroid receptor coactivator (Src), mainly involved in migratory and invading mechanisms^8^. Consistent with these data, several studies demonstrated a role for *PAK1* genomic alterations (i.e., amplification) and over-expression in enhancing cell motility, invasiveness, and anchorage-independent growth of epithelial cells, promoting metastatic properties^9^. Moreover, Pak1 kinase increased activity correlated to invasive phenotype in breast cancer cells^6^.

In this study, we generated two ER+/HER2- breast cancer cells resistant to the combination of fulvestrant (ER down regulator) and abemaciclib (CDK4/6i). Fulvestrant and abemaciclib resistant cells (FAR cells) exhibited an invasive phenotype. Pak1 resulted hyper-activated in our FAR models compared to parental cells prompting us to investigate its role in the resistant phenotype. Our results suggest a crucial part of Pak1 as a mediator of resistance to antiestrogens *plus* CDK4/6 inhibitors. Interestingly, we showed that Pak1 inhibition restored cell sensitivity to fulvestrant and abemaciclib in FAR cells in vitro and in vivo, hampering cell viability, invasion, and anchorage-independent growth.

## Results

### ER+ breast cancer fulvestrant-abemaciclib resistant (FAR) cells show an increased proliferative and invasive phenotype compared to parental cells

**Fig. 1:**
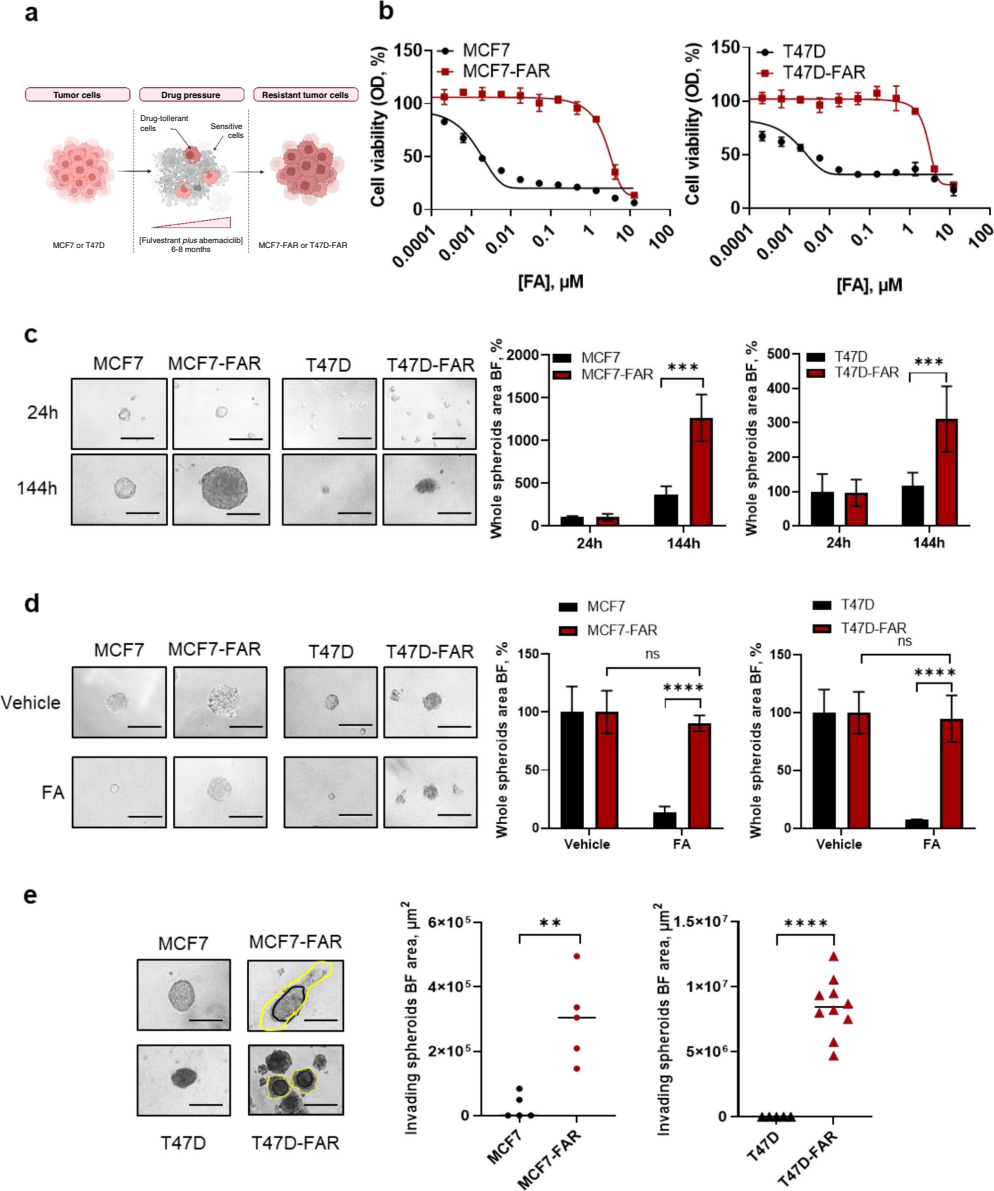
Generation and characterization of fulvestrant and abemaciclib resistant ER+ breast cancer cell lines. Schematic representation of fulvestrant-abemaciclib resistant cells (FAR) generation (**a**). Dose-response curves of MCF7, MCF7-FAR (left) or T47D, T47D-FAR (right) exposed to increasing doses of fulvestrant and abemaciclib (FA; up to 10 µM fulvestrant +2.5 µM abemaciclib) combination every 72 h for 1 week and stained with crystal violet. Each data point represents the percent of viable cells relative to vehicle-treated controls (**b**). Representative images of spheroids from 5 × 10^4^ MCF7 and MCF7-FAR or 10 × 10^4^ T47D and T47D-FAR cultured up to 144 h in ultra-low attachment plates (left). All images were capture at 20x magnification (Bars = 200 µm). Spheroids area is reported in the right panels for MCF7/MCF7-FAR and T47D/T47D-FAR. Values are expressed as percentage relative to the area calculated 24 h after spheroid formation (**c**). Representative images of spheroids from 5 × 10^4^ MCF7 and 2.5 × 10^4^ MCF7-FAR, 10 × 10^4^ T47D and 5 × 10^4^ T47D-FAR spheroids exposed to 400 nM fulvestrant and 100 nM abemaciclib (FA) every 72 h for 7days (left). Magnification 20x (scale bar = 200 μm). On the right, bar graphs showing percentage of whole spheroids area upon FA treatment compared to spheroids treated with vehicle (plotted as 100%) (**d**). Representative images of MCF7 and MCF7-FAR spheroids or T47D and T47D-FAR spheroids (left) embedded in collagen type I matrix for 7 days. All images were capture at 20x magnification (Bars = 200 µm) (**e**). Quantification of invading area (yellow lines), calculated by subtracting the core area (black lines) to the total spheroidal one, is reported in right panel for both MCF7/MCF7-FAR and T47D/T47D-FAR. For all panels data are expressed as mean±standard deviation (SD) of three separate experiments, indicated by error bars, performed in triplicate or quadruplicate (^**^p < 0.01; ^***^p < 0.001; ^****^p < 0.0001; *Student’s T-test*).

MCF7 and T47D cells with low-to-none sensitivity to fulvestrant and abemaciclib (hereafter referred to as MCF7-FAR and T47D-FAR, respectively) were generated to investigate resistance mechanisms to antiestrogens and CDK4/6i (Fig. 1a). To this purpose, MCF7 and T47D cells were chronically treated with increasing doses of both drugs until less responsiveness compared to parental cells was obtained (up to 1 µM of fulvestrant and 0.25 µM of abemaciclib). Dose-response curves demonstrated that MCF7-FAR and T47D-FAR cells were less sensitive to fulvestrant and abemaciclib as single agents (Supplementary Fig. 1a, b) and to the combination of both drugs compared to parental cells (Fig. 1b). Western blot analysis showed loss of Rb expression in MCF7-FAR cells compared to parental cells (Supplementary Fig. 1c), unlike T47D-FAR cells that maintained Rb expression (Supplementary Fig. 1c). Moreover, we found reduced ERα levels in MCF7-FAR and T47D-FAR cells compared to parental cells (Supplementary Fig.1c), suggesting a potential switch from a luminal to a more invasive phenotype. Indeed, spheroids formation analysis revealed that MCF7-FAR and T47D-FAR cells showed an increased ability to grow in an anchorage-independent manner in ultra-low attachment conditions compared to parental cells, resulting from the analysis of tumor spheroids area over time in absence of drug treatments (Fig. 1c).

Moreover, treatment with fulvestrant and abemaciclib almost completely prevented MCF7 and T47D cell growth (Fig. 1d). Instead, MCF7-FAR and T47D-FAR – set up to obtain a similar spheroids area between vehicle-treated parental and FAR - showed a blind decrease in spheroids’ formation after combined treatment with fulvestrant and abemaciclib (Fig. 1d).

Invasive transwell-based assays showed a higher capability of MCF7-FAR and T47D-FAR cells to invade compared to MCF7 and T47D, respectively (Supplementary Fig. 2a). In agreement with these results, MCF7-FAR and T47D-FAR spheroids embedded in a collagen I matrix showed a significant increase in tumor spheroids invasion in 3 dimensional (3D) conditions compared to MCF7 and T47D, respectively (Fig. 1e). The expression of cytoskeleton protein F-actin, as well as the assembly and disassembly of actin filaments, has been shown as a canonical biomarker of cell migration and invasion, crucial for cell movement^10^. Hence, we performed phalloidin staining in MCF7/MCF7-FAR and in T47D/T47D-FAR. Our analysis revealed an increased expression of the cytoskeleton protein F-actin and an increase in actin-fibers re-arrangement in FAR cells compared to parental cell lines (Supplementary Fig. 2b, c), a crucial mechanism enabling tumor cell migration and invasion^11,12^.

### RNA-Sequencing data reveal p21-activated kinase 1 (Pak1) as a putative mediator of resistance to fulvestrant and abemaciclib in ER+ breast cancer cells

**Fig. 2:**
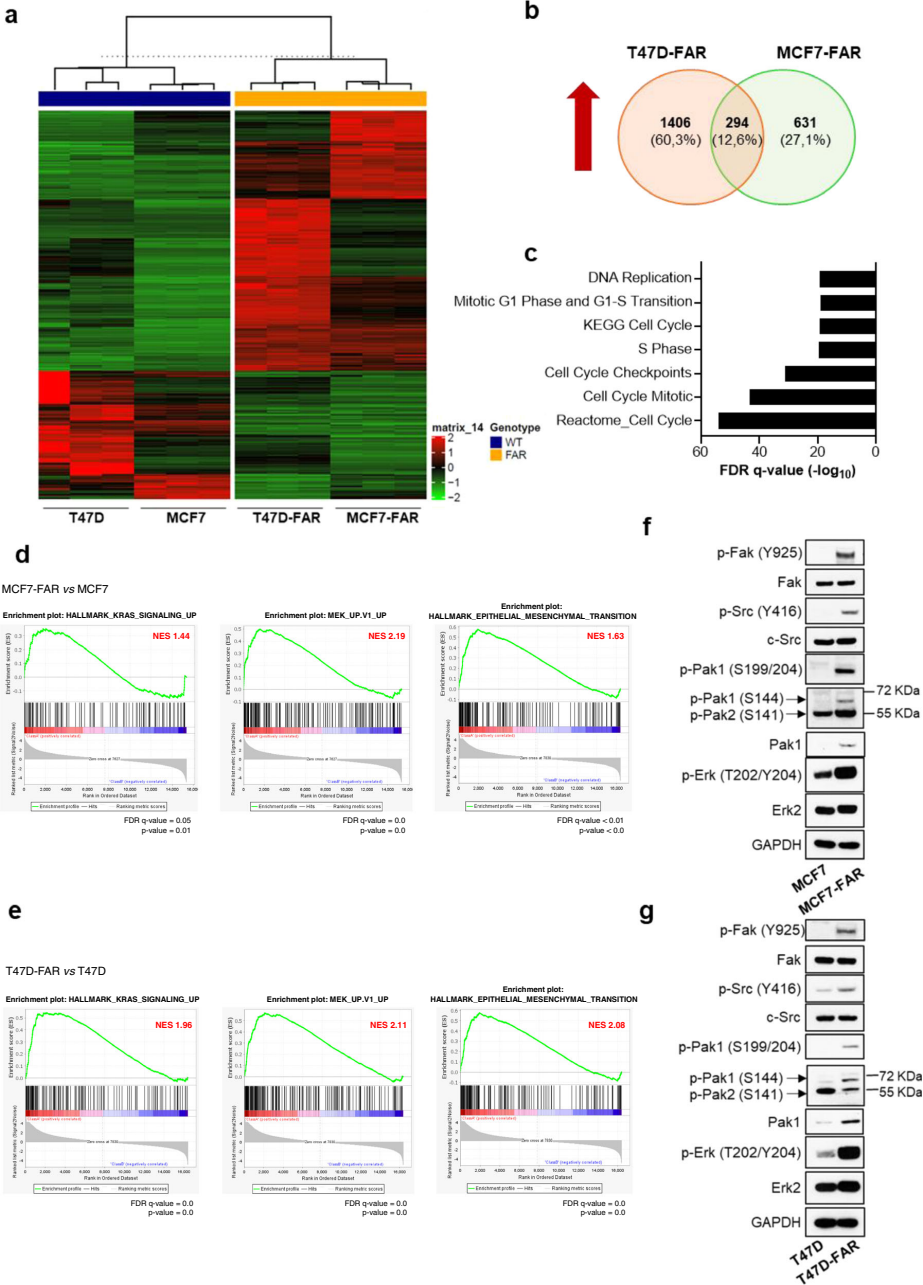
Evaluation of the common transcriptional reprogramming underpinning drug resistance to fulvestrant and abemaciclib in ER+ breast cancer cell lines. Heatmap of hierarchical clustering indicating differentially expressed genes (rows) between parental and FAR cells (**a**). Venn-diagram of common up-regulated genes between MCF7-FAR and T47D-FAR is shown in (**b**). Bar graphs showing gene set names obtained by Gene Set Enrichment Analysis (GSEA) of common 294 up-regulated genes between MCF7-FAR and T47D-FAR cells. Data are plotted for False Discovery Rate (FDR) *q*-value (**c**). RNA-seq based GSEA of pathways significantly up-regulated in FAR resistant *vs* parental MCF7 and T47D cells are shown with Normalized Enriched Score (NES) and FDR *q*-value and *p*-value (**d**, **e**). Western blot analysis of sensitive MCF7 and T47D or resistant FAR collected after 18 h of treatment with fulvestrant (1 µM) and abemaciclib (0.25 µM) (**f**, **g**, respectively). Whole cell lysates were prepared and subjected to immunoblot analysis with the indicated antibodies. Images are representatives from three independent experiments.

In order to identify common mechanisms of resistance to ET and CDK4/6i in both MCF7-FAR and T47D-FAR, we performed a transcriptomic analysis by RNA-Sequencing of parental and resistant cells in presence of fulvestrant and abemaciclib. The analysis revealed a specific transcriptional reprogramming of MCF7-FAR and T47D-FAR cells compared to MCF7 and T47D cells, respectively, as shown in Fig. 2a. The intersection of differentially expressed genes in MCF7-FAR and T47D-FAR identified 294 common up-regulated genes (Fig. 2b). The analysis of canonical pathways revealed that common hyper-expressed genes are involved in cell growth and cell cycle progression, further supporting the phenotypic observation of the less responsiveness of FAR cells to fulvestrant and abemaciclib (Fig. 2c). Moreover, gene-set enrichment analysis (GSEA) showed significant enrichment of KRAS, MEK, and epithelial to mesenchymal transition (EMT) pathways in both MCF7-FAR and T47D-FAR compared to the respective parental cells (Fig. 2d, e). To validate the activation of these pathways, we performed a western blot analysis that revealed an increase in the phosphorylation levels of Erk signaling (a key mediator of MEK/ERK axis) as well as of Fak and Src (crucial kinases involved in motility and invasion pathways) (Fig. 2f, g). Thus, we sought a common intra-cellular mediator with an active role in cell migration and proliferation, whose inhibition might reverse not only resistance to ET and CDK4/6i, but also might prevent FAR high invasive phenotype. Interestingly, we found a significant and robust increase in p21-activated kinase 1 (Pak1) activation and expression in MCF7-FAR and T47D-FAR compared to MCF7 and T47D, respectively, prompting us to investigate its role in drug resistance (Fig. 2f, g; Supplementary Fig. 3a, b).

### *PAK1* over-expression reduces tumor cell sensitivity to fulvestrant and abemaciclib in ER+ breast cancer cell lines

**Fig. 3:**
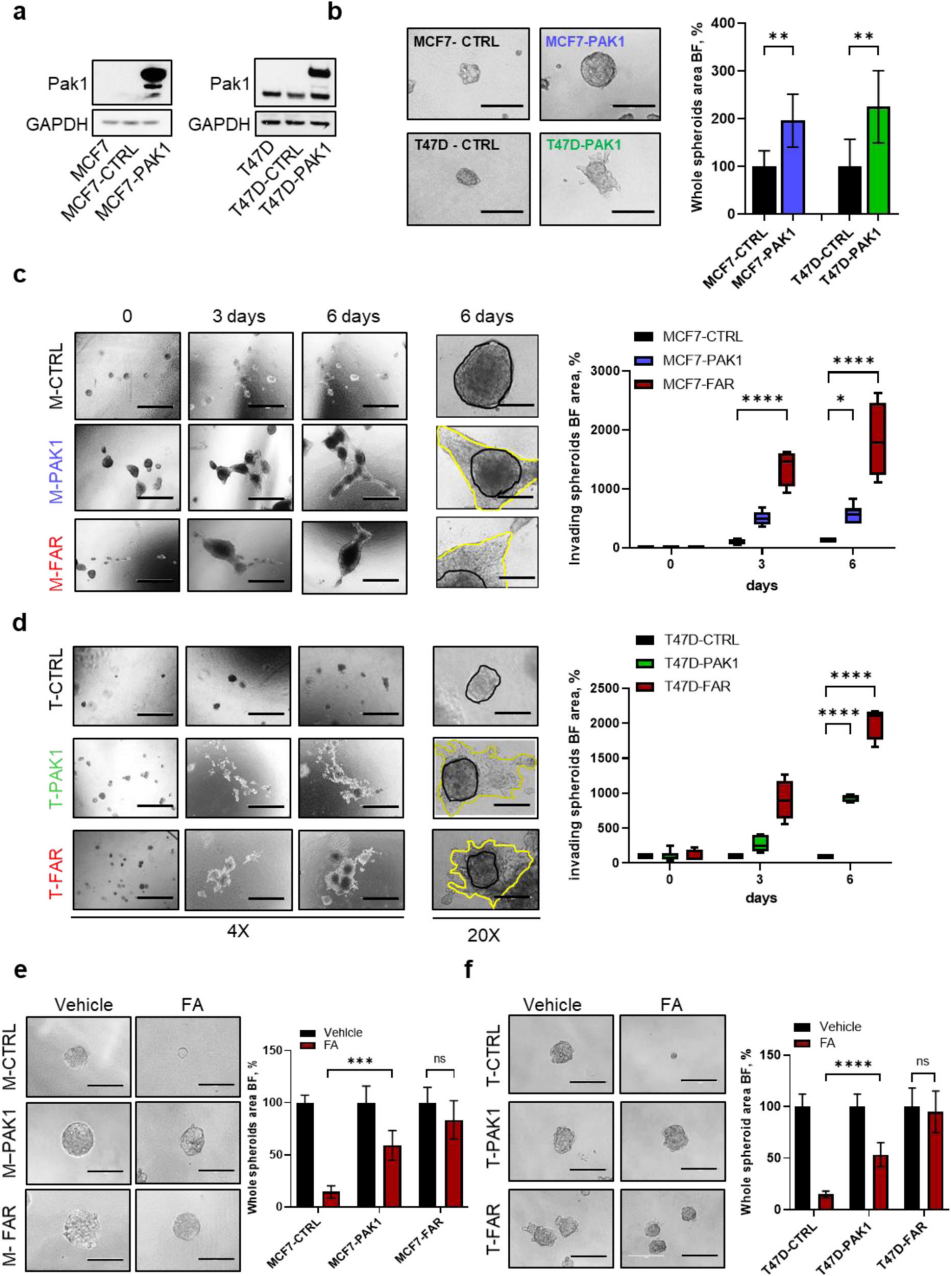
Effects of *PAK1* over-expression on cell proliferation and invasion in MCF7 and T47D cells. Representative western blot analysis for Pak1 of parental MCF7 (left) and T47D (right), GFP vector control (pLenti-C-mGFP-P2A-Puro) over-expressing cells (MCF7-CTRL and T47D-CTRL) or *PAK1*-over-expressing cells (MCF7-PAK1 and T47D-PAK1). GAPDH was used as a loading control (**a**). Representative images of MCF7-CTRL or -PAK1 and T47D-CTRL or -PAK1 (left) spheroids cultured for 72 h. All images were capture at 20x magnification (Bars = 200 µm). Bar graph shows the whole spheroids area plotted as percentage relative to spheroids CTRL area (right) (**b**). Representative images of spheroids from MCF7-CTRL or -PAK1 or -FAR cells (**c**) and T47D-CTRL or -PAK1 or -FAR cells (**d**) embedded into a collagen type I matrix at different time points. All images were capture at 4x magnification (Bars = 1000 µm), or 20x magnification (scale bar = 200 μm). Box plots showing spheroid invading area are reported for MCF7 (**c**, right) and T47D (**d**, right) models. Data are plotted with median and SD. Representative images of MCF7-CTRL, -PAK1 or –FAR (**e**, left) and T47D-CTRL, -PAK1 or –FAR (**f**, left) spheroids treated for 72 h with vehicle or with the combination of 400 nM of fulvestrant and 100 nM of abemaciclib (FA). Magnification 20x (scale bar = 200 μm). Bar graphs showing percentage of whole spheroids area upon FA treatment compared to spheroids treated with vehicle (plotted as 100%) are reported for MCF7 (**e**, right) and T47D (**f**, right) models. For panels **b**, **e**, **f** right, data are expressed as mean ± SD performed in quadruplicate. For al panels (^*^p < 0.05; ^**^p < 0.01, ^***^p < 0.001; ^****^p < 0.0001; 2way ANOVA Bonferroni’s multiple comparisons).

To assess Pak1 role as a potential mediator of resistance to fulvestrant and abemaciclib, we over-expressed *PAK1* in parental MCF7 and T47D (Fig. 3a). *PAK1* over-expressing cells significantly improved cell growth ability in anchorage-independent manner, resulting from tumor spheroids’ area analysis reported in Fig. 3b in both cell lines. Moreover, *PAK1* over-expression in MCF7 and T47D cells significantly enhanced invasion, mimicking the acquired invasive phenotype of FAR models, as revealed by the number of invading cells able to penetrate matrigel matrix in transwell inserts and by tumor spheroids invading area measured in a collagen type I matrix (Supplementary Fig. 4a, b; Fig. 3c, d). Finally, MCF7-PAK1 and T47D-PAK1 over-expressing cells showed – similar to FAR cells - a significant increase in tumor spheroids growth in presence of fulvestrant and abemaciclib compared to MCF7 and T47D expressing control vector, respectively (Fig. 3e, f). Accordingly, dose-response curves clearly showed that MCF7-PAK1 and T47D-PAK1 were less sensitive to increasing doses of fulvestrant and abemaciclib combination, compared to parental MCF7 and T47D, respectively, partially recapitulating resistance of FAR cells (Supplementary Fig. 4c, d).

### *PAK1* down-regulation restores drug sensitivity to fulvestrant and abemaciclib in FAR cells

**Fig. 4:**
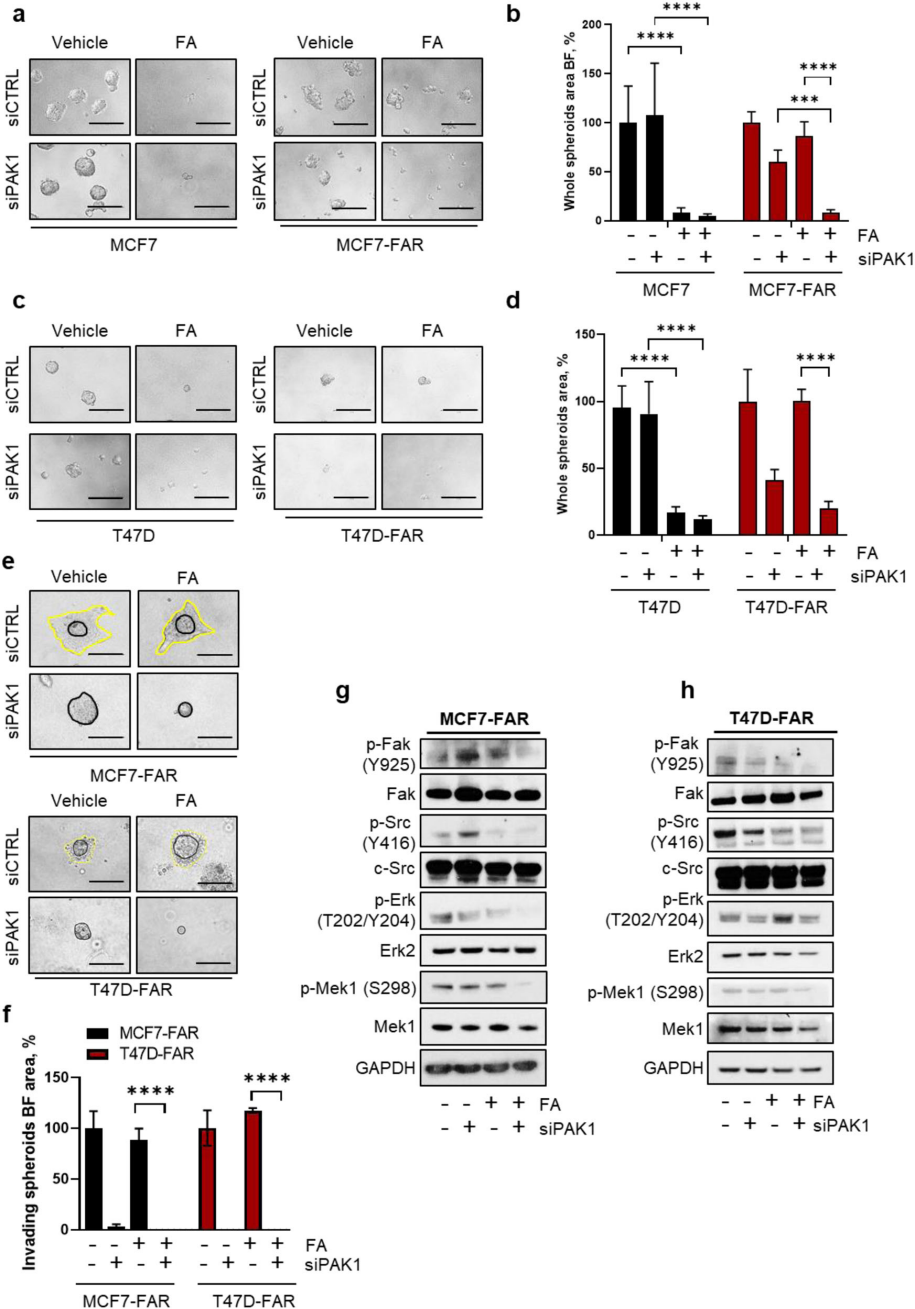
*PAK1* knock-down as key event in increasing resistant cell sensitivity to fulvestrant and abemaciclib. Representative images of MCF7 (**a**, left) and MCF7-FAR (**a**, right) or T47D (**c**, left) and T47D-FAR (**c**, right) spheroids transfected with siRNA scrambled (siCTRL) or siRNA targeting *PAK1* (siPAK1) for 72 h and treated with vehicle or the combination of 400 nM of fulvestrant and 100 nM of abemaciclib (FA) for other 72 h. Magnification 20x (scale bar = 200 μm). Bar graphs showing percentage of whole spheroids area upon FA treatment ± si*PAK1* compared to spheroids treated with vehicle (plotted as 100%) are reported for MCF7 and MCF7-FAR (**b**) and T47D and T47D-FAR (**d**) cells **(a**–**d)**. Representative images of spheroids from MCF7-FAR (**e**, top) and T47D-FAR (**e**, bottom) knocked-down for *PAK1* and treated with vehicle or the combination of 400 nM fulvestrant and 100 nM abemaciclib (FA) embedded in collagen type I matrix for 6 days. Magnification 20x (scale bars = 200 μm). Bar graph showing quantification of invading spheroids area upon silencing of *PAK1* and in presence or not of FA (**f**). Data are plotted as percentage relative to cells transfected with scrambled siRNAs and treated with vehicle (**f**). Representative western blot analysis for the indicated antibodies of MCF7-FAR (**g**) or T47D-FAR (**h**) total lysates of cells silenced for *PAK1* for 72 h and treated for further 72 h with 1 μM of fulvestrant and 0.25 μM of abemaciclib (FA). GAPDH was used as loading control. Images are representatives of three independent experiments. For all panels, data are plotted as means±SD of three independent experiment performed in quadruplicate (^***^p < 0.001; ^****^p < 0.0001; 2way ANOVA Bonferroni’s multiple comparisons).

**Fig. 5:**
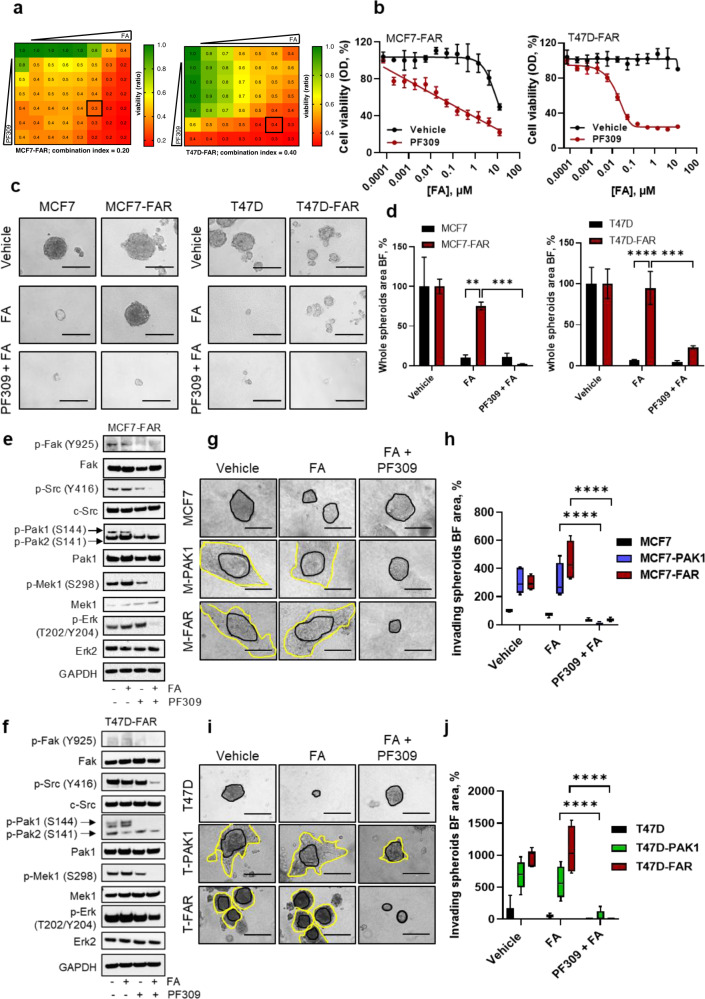
Impairment of FAR cell growth and invasion upon Pak1 pharmacological inhibition. Viability assay to test synergy between FA and PF309. Cells were treated with increasing concentrations of fulvestrant and abemaciclib (FA) and PF309 (up to 10 + 2.5 μM and 0.1 μM, respectively) alone or in combination every 72 h until vehicle-treated controls reached ∼90% of confluence. Intensity values of cell monolayers stained with crystal violet were used to perform the Chou-Talalay test. Numbers inside each box indicate the *ratio* of viable treated cells to untreated cells from three independent experiments for MCF7-FAR (**a**, left) and for T47D-FAR (**a**, right). Dose-response curves of MCF7-FAR (**b**, left) or T47D-FAR (**b**, right) exposed to increasing doses of fulvestrant and abemaciclib (FA), in presence or not of 10 nM of PF309 for 1 week. Representative images of MCF7 and MCF7-FAR (**c**, left) or T47D and T47D-FAR (**c**, right) spheroids exposed to 400 nM fulvestrant and 100 nM abemaciclib (FA) ± 10 nM PF-309. Bar graphs showing percentage of whole spheroids area upon FA ± PF-309 treatment compared to spheroids treated with vehicle (plotted as 100%) are reported for MCF7/MCF7-FAR (**d**, left) and T47D/T47D-FAR (**d**, right) cells. Western Blot analysis of MCF7-FAR (**e**) and T47D-FAR (**f**) treated with fulvestrant and abemaciclib (FA, 1 μM and 0.25 μM, respectively), PF-309 (500 nM) or the combination for 48 h. GAPDH was used as loading control. Images are representatives from three independent experiments. Morphology of MCF7, MCF7-PAK1 and MCF7-FAR (**g**) or T47D, T47D-PAK1 and T47D-FAR (**i**) spheroids cultured into a collagen matrix in presence of vehicle or 400 nM fulvestrant and 100 nM abemaciclib (FA) ± 10 nM PF-309. All images were capture at 20x magnification (Bars = 200 µm). Box plot showing spheroids invading area is reported for MCF7 and T47D models (**h**, **j** respectively). Data are plotted relative to vehicle-treated MCF7 **(h)** and T47D **(j)** area. Middle line in box represents the median±SD performed in quadruplicate (^****^p < 0.0001, 2way ANOVA Bonferroni’s multiple comparisons). For panel **d**, data are plotted as means ± SD of three independent experiment performed in triplicate or quadruplicate (^**^p < 0.01; ^***^p < 0.001; ^****^p < 0.0001; 2way ANOVA Bonferroni’s multiple comparisons).

We explored whether *PAK1* gene silencing restored sensitivity to FA in FAR cells. siRNA-mediated *PAK1* down-regulation in FAR cells was verified by qPCR after 24 h of transfection (Supplementary Fig. 5a). si*PAK1* led to 30% decrease in MCF7-FAR and to 60% decrease in T47D-FAR tumor spheroids area (Fig. 4a–d). Spheroids growth was almost completely abolished in presence of fulvestrant and abemaciclib, demonstrating that *PAK1* down-regulation is required to restore sensitivity to FA in FAR cells (Fig. 4a–d). In contrast, in parental Pak1 low-expressing MCF7 and T47D cells, *PAK1* knock-down exerted no effects on spheroids growth (Fig. 4a–d), which in turn was affected by the only treatment with FA. Moreover, tumor invasion, evaluated through matrigel-coated transwell inserts and spheroids’ invading area, was not impaired in parental not-invasive MCF7 and T47D (Supplementary Fig. 5b) while was strongly altered by *PAK1* down-regulation in MCF7-FAR and T47D-FAR (Fig. 4e, f; Supplementay Fig. 5c, d). These results indicated FA and Pak1 targeting combination as crucial to abolish both tumor proliferation and invasion. Consistent with phenotypic assays, combination of fulvestrant and abemaciclib treatment with si*PAK1* in MCF7-FAR and T47D-FAR cells induced a decrease in both p-Erk and p-Mek levels, as well as in p-Fak and p-Src levels (Fig. 4g, h), confirming that *PAK1* down-modulation might prevent both the activation of proliferative and invading pathways.

### Pharmacological treatment with Pak1 inhibitors restores sensitivity to fulvestrant and abemaciclib in FAR cells both in vitro and in vivo

To further verify that Pak1 inhibition could circumvent resistance to FA, we pharmacologically inhibited the kinase by using PF-03758309 (PF309) and NVS-PAK1-1, a potent ATP-competitive inhibitor of Pak kinase family with a high affinity to Pak1 and a specific inhibitor of Pak1, respectively^13,14^. Firstly, we assessed drug interaction between Pak inhibitors and FA using Chou-Talalay method^15^, treating MCF7-FAR and T47D-FAR with increasing FA and PF309 or NVS-PAK1-1 (Fig. 5a; Supplementary Fig. 6a). The combination studies clearly showed synergy between these agents, successfully preventing tumor cell growth (CI = 0.20 and CI = 0.28 for MCF7-FAR with PF309 and NVS/PAK1-1; respectively; CI = 0.40 and CI = 0.29 for T47D-FAR with PF309 and NVS/PAK1-1, respectively; Fig. 5a; Supplementary Fig. 6a). In accordance, dose-response curves showed that the addition of PF309 or NVS-PAK1-1 to FA in both MCF7-FAR and T47D-FAR cells affected cell viability (Fig. 5b; Supplementary Fig. 6b). Spheroids formation analysis further demonstrated that PF-309 and NVS-PAK1-1 sensitized FAR cells to endocrine therapy and CDK4/6i (Fig. 5c, d; Supplementary Fig. 6c, d). The effect of Pak1 inhibitors on cell growth was associated with a robust decrease in Mek, Erk, Fak, and Src phosphorylation levels, further suggesting a potential anti-proliferative and anti-invasive effect of the triple-drug combination in both MCF7-FAR and T47D-FAR cells (Fig. 5e, f; Supplementary Fig. 6e). Moreover, the higher invasive capabilities of MCF7-PAK1 or T47D-PAK1 and MCF7-FAR or T47D-FAR cells were successfully reverted upon PF309 and NVS-PAK1-1 treatments (Supplementary Fig. 7a–d; Fig. 5g–j).

**Fig. 6:**
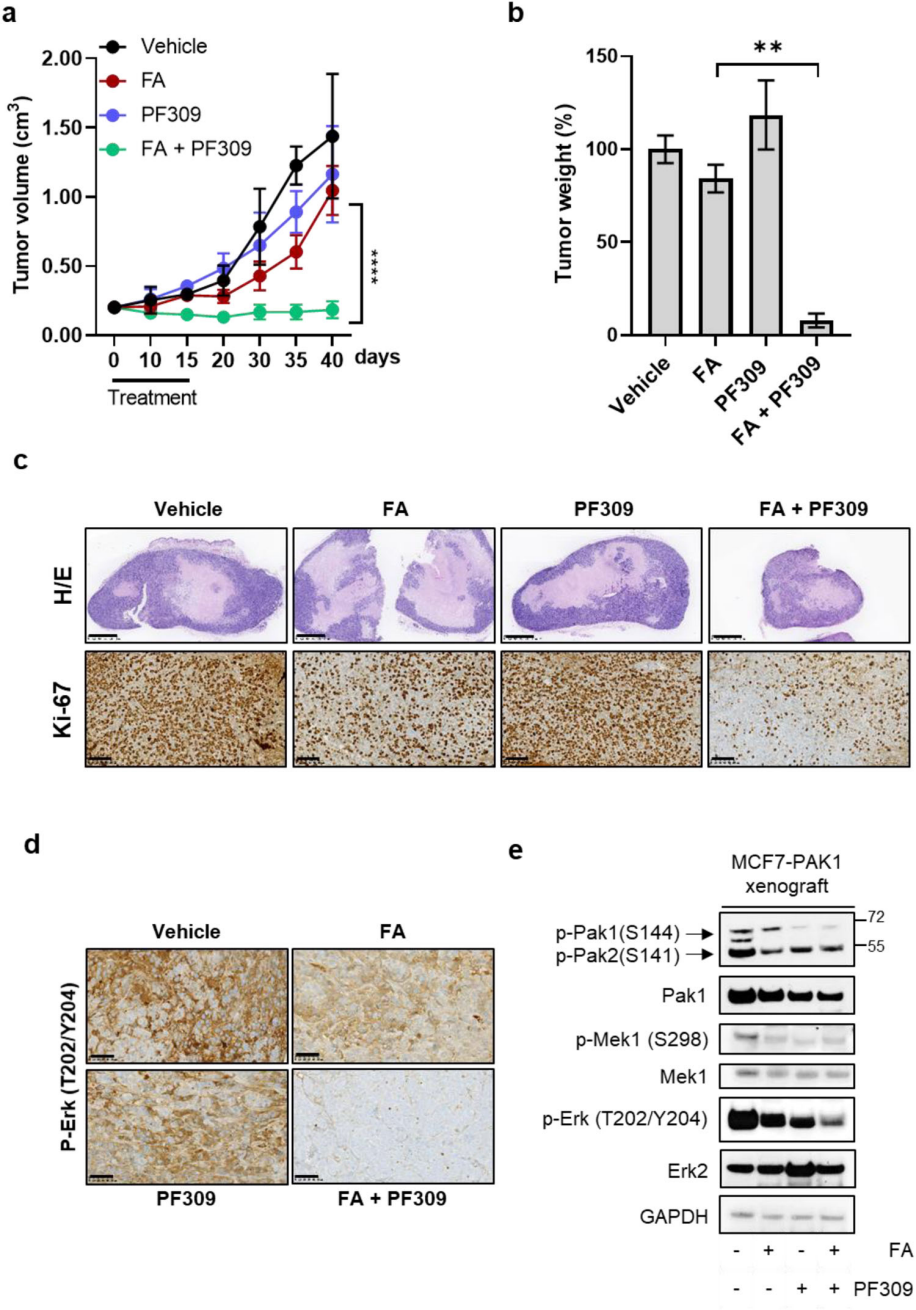
Tumor xenograft growth impairment and drug resistance reversion upon Pak1 pharmacological inhibition. MCF7-PAK1 xenografts were established in Balb/c nude mice. Once tumors reached ≥200 mm^3^, mice were randomized to treatment with vehicle, fulvestrant and abemaciclib (FA; 5 mg/week, 25 mg/kg p.o., respectively), PF309 (15 mg/kg p.o.) alone or in combination. Tumor growth curve is showed in **a**. Each data point represents the mean of tumor volume in cm^3^ ±SD (*n* = 5 per arm, ^****^p < 0.0001 *vs*. FA drug arms; *Student’s t-test*). A*t* the end of the treatment tumours were collected, weights were measured and reported in bar graph as percentage relative to vehicle-treated control (**b**). Representative images of MCF7-PAK1 xenografts’ sections stained with Hematoxylin and eosin (H/e) or processed for Ki67 staining are showed in **c** (top panels and bottom panels, respectively). Images were capture at 2.5x (top panels) or 20x magnification (bottom panels); bars=1000 µm or 100 µm, respectively. Representative images of MCF7-PAK1 xenografts’ sections subjected to IHC for p-ERK (T202/Y204) **(d)**. All images were capture at 20x magnification (Bars=100 µm). Western blot analysis of proteins extracted from MCF7-PAK1 tumor xenografts after 4 h of treatment and blotted for the indicated antibodies (**e**). Images are representatives from three independent experiments.

Finally, to assess the impact of Pak1 targeting in tumoral response to ET *plus* CDK4/6i in vivo, MCF7-PAK1 cells were injected in Balb/c nude mice. After four weeks, mice were randomized to treatment with vehicle, fulvestrant *plus* abemaciclib, PF309, PF309 +fulvestrant *plus* abemaciclib. Compared to treatments with fulvestrant and abemaciclib that only slightly delayed tumor growth, the combination treatment of FA and PF309 significantly prevented tumor growth (Fig. 6a, b), without any significant body weight loss (Supplementary Fig. 8a). 2-weeks course of triple treatment was associated with a decrease of two proliferative tumor markers, as Ki67 and phosphorylation of Erk, in MCF7-PAK1 xenografts (Fig. 6c, d). In accordance, western blot analysis showed a concurrent down-regulation of p-Mek and p-Erk phosphorylation (Fig. 6e), supporting the evidence of lower proliferative potential compared to tumors treated with only FA or PF-309.

In conclusion, our evidence suggests that the aberrant activation of Pak1 might play a key role in resistance to endocrine therapy and CDK 4/6i in ER+ breast cancer cells. Our data showed that Pak1 abrogation can affect not only cell growth but also tumor-resistant cell invasion. Thus, the combination of Pak1 inhibitors to fulvestrant and abemaciclib might represent a valid therapeutic option for selected patients who progressed after endocrine and CDK4/6i combined therapy.

## Discussion

The use of CDK4/6 inhibitors in combination with endocrine therapy represents one of the most impactful therapeutic advances that radically improved survival of patients with breast cancer^16^. The dissection of molecular mechanisms underlying resistance to therapies remains one of the main goals of oncologic research. Several mechanisms of resistance to CDK4/6i have been described so far, including alterations of cell-cycle-related genes or abnormalities of tyrosine kinase receptors (RTKs) and pathways^17^. For instance, *Rb* loss, amplification and over-expression of *CDK6*, as well as activating mutations in *CDK4*, have been shown to facilitate cell cycle progression, conferring resistance to CDK4/6i^18,19^. Focusing on non-cell cycle-related genes, *FGFR1* amplification led to low sensitivity to CDK4/6i^20^ and ET by influencing gene transcription^21^. Similarly, *RAS* and *AKT1* activating alterations have been detected in liquid biopsies of patients that progressed after CDK4/6 inhibition^22^.

The current study suggests Pak1 as a therapeutic target to overcome resistance to endocrine therapy and CDK4/6i. Briefly, we demonstrated that ER+ breast cancer cell lines resistant to the combination of fulvestrant and abemaciclib (MCF7-FAR and T47D-FAR) developed an increased anchorage-independent growth and invasive phenotype. These cell lines showed higher Pak1 expression and phosphorylation levels than sensitive cells, prompting us to investigate the functional role of Pak1 in the onset and maintenance of tumor resistance. Pak1 expression in breast cancer positively correlates to metastasis and poor prognosis^23,24^. In particular, *PAK1* over-expression and/or amplification have been demonstrated to promote both development and progression of different cancers^3,25–28^, mainly impairing growth-signalling networks (i.e., MAPK). In luminal A breast cancers Dang et al., found that high expression of Pak1 significantly correlated to worse clinical outcomes^29^.

Additionally, Pak1 activity can also orchestrate the anchorage-independent growth by regulating Cdc42/Rac activation by Fak/Src complex^30^. Thus, Pak1 directly fine-tunes focal adhesion dynamics leading to a motile phenotype, can promote epithelial-to-mesenchymal transformation by inducing higher expression of matrix metalloproteinase genes and *via* directly activating transcription factors^6,31,32^ and can augment proliferation mediating Erk phosphorylation^9^. Consistent with these data, in our model *PAK1* over-expression and hyper-activation triggered MAPK pathway and Fak kinase - which activates Src^33^ - ultimately pushing proliferation and invasion of FAR cells. Similar to our system, in metastatic melanoma cells characterized by *BRAF* mutations, resistance to MEK and BRAF inhibitors is mediated by MAPK rebound, and more specifically, Erk re-activation is mediated by Pak1^34^.

In addition to Pak1 involvement in tumor progression, its role in drug resistance has already been described in multiple cancers^35,36^. For instance, Pak1 inhibition remarkably prevented also tumor growth and metastasis formation in breast cancer and re-sensitized resistant cells to tamoxifen^37^.

Our study further supports the hypothesis that targeting an intracellular mediator of growth and motility-related pathways – rather than RTKs as usually suggested in breast cancer–could represent a good option to affect the main aberrant signalings underpinning resistance to FA. Our work, indeed, revealed that Pak1 abrogation by siRNA or pharmacological inhibitors (PF-309 / NVS-PAK1-1) in combination with fulvestrant and abemaciclib significantly abolished the activation of MEK/ERK and Fak proteins, in turn preventing FAR cell proliferation and invasion and thus overcoming resistance in vitro and in MCF7-PAK1 xenograft models. The effects of PF-309 and NVS-PAK1-1 on proliferation and invasive properties of FAR cells further suggested a role of Pak1 in resistant phenotype. These results provide a rationale for further clinical development for Pak inhibitors in ER+ breast cancers. Indeed, the dual effect of Pak1 mediation on two critical hallmarks of cancer (proliferation and invasion) may represent an effective targeting that could robustly improve the response to therapies. In order to validate Pak1 as a marker of acquired mechanisms of resistance also in patients’ cohort, the evaluation of *PAK1* expression might be investigated in future studies in tumor biopsies of patients with disease progression after ER+ *plus* CDK4/6i therapy.

In conclusion, our study provides new insights into resistance mechanisms to CDK4/6i *plus* endocrine therapy, shedding light on compensatory signalling pathways that could strongly compromise the percentage of responders’ patients. Based on our results, we propose Pak1 inhibitors combined with ET and CDK4/6 inhibitors as a possible approach for the treatment of ER+ resistant breast cancers.

## Methods

### Cell lines and inhibitors

MCF-7 (ATCC® HTB-22™), T47D (ATCC® HTB-133™) human breast cancer cells were obtained from the American Type Culture Collection (ATCC) and maintained in ATCC-recommended media supplemented with 10% FBS (Gibco) and 1× antibiotic/antimycotic (Gibco). MCF7-FAR, T47D-FAR cells were generated after 6 months of treatment with increasing doses of abemaciclib in combination with fulvestrant up to 0.25 µM or 1 µM final concentration, respectively. MCF7-PAK1 and T47D-PAK1 cell lines were generated after lentiviral infection (lentiviral vector: pLenti-C-mGFP-P2A-Puro-PAK1) of parental MCF7 or T47D, respectively, as described below. Cell lines were authenticated by ATCC prior to purchase by the short tandem repeat (STR) method. All experiments were performed <2 months after thawing of early passage cells. Mycoplasma testing was conducted for each cell line before use.

Fulvestrant was purchased from SelleckChem (Houston, TX, USA), abemaciclib was provided by Eli Lilly and Company (Indianapolis, IN, USA), PF-03758309 was provided by Pfizer Inc. (New York, NY, USA), NVS-PAK1-1 was purchased from SelleckChem (Houston, TX, USA).

### Breast cancer Spheroids assay

5 × 10^3^ parental, FAR or PAK1-overexpressing MCF7 or T47D were seeded in quadrupled in Ultra-Low attachment 96 plate (#CLS3474, Corning) in 100 µL of 10% DMEM-FBS for 48 h. After that, representative images were captured at InvitrogenTM EVOSTM FL imaging system (20X magnification), spheroids were treated for 72 h as detailed in the specific figure legends. Tumor spheroids growth was monitored at the inverted microscope, the area was quantified with the ImageJ 1.53 software (NIH, Bethesda, MD, USA) and normalized to time 0 area. Spheroids from MCF7 or T47D parental, or FAR transfected with siCTRL or siPAK1 were set up after 48 h from the siRNA transfection. Then, they were treated and the area was measured as described in the figure legends.

### 3D spheroid and transwell-based invasion assay

5 × 10^3^ parental or FAR MCF7 or T47D cells transfected with siRNA CTRL or against *PAK1*, were seeded in octoplicate in Ultra-Low attachment 96 plate in 100 µL of 10% DMEM-FBS for 4 days. Tumor spheroids from 4 wells were collected, centrifuged at 1200 rpm for 5 min and resuspended in 100 µL FBS for each collagen matrix. Spheroids were embedded in a neutralized collagen I solution at 2 mg/mL final concentration (#C3867, Sigma-Aldrich) and seeded in 24 well plate. Samples were treated with the indicated drugs for 6 days, every 72 h, as detailed in the specific figure legends. Spheroids growth and invasion was monitored at the inverted microscope, the whole and invading spheroids area were quantified with ImageJ 1.53 software (NIH, Bethesda, MD, USA). The invading spheroids area was calculated by subtracting the area of spheroidal nucleus from the total spheroidal surface, normalizing to time 0 area.

For 2D invasion assays, 7 × 10^4^ cells were seeded into the top chamber of transwell (pore size, 8 mm; Corning) pre-coated with Matrigel (Corning) in DMEM: Matrigel ratio 1:1. After 6 days, invading cells were stained in 0.1% crystal violet for 30 min. Invading cells were counted and plotted as mean of cell count *per* field.

### siRNA transfections

Cells were transfected using Lipofectamine RNAi-MAX® (Invitrogen) and 20 nM ON-TARGETplus Non-targeting Pool (#D-001810-10-20, Dharmacon) or SMARTpool: ON-TARGETplus *PAK1* siRNA, (#L-003521-00-0020, Dharmacon) at 40 nM final concentration. After two days, cells were seeded in 10% DMEM-FBS in either ultra-low attachment 96-well plates (5 × 10^3^/well for spheroids assays) or in 60 mm plates (for immunoblot analysis) and treated at the indicated drug concentration for 72 h. For immunoblot analyses, cells were harvested and total cell lysates prepared 3 days after siRNA transfection and 3 more days after drug treatment. Representative images of tumor spheroids were captured at InvitrogenTM EVOSTM FL imaging system (20X magnification) the day after seeding and 6 days after drug treatment (media±drug were changed every 72 h).

### Viral transduction

Human mGFP-tagged-PAK1 (RC225947L4V) and pLenti-C-mGFP control (PS100071V) lenti-ORF particles were purchased from OriGene. To generate stably transduced cell lines, 25 µL of lenti-ORF particles were transfected with 8 μg/mL polybrene (Sigma Aldrich) in MCF7 or T47D parental cells. After 48 h, transduced cells were selected in 1 μg/mL puromycin.

### RNA extraction, RT-PCR, qPCR

RNA was isolated using TRIzol and 1 μg of RNA/sample was reverse-transcribed using SuperScript™ III Reverse Transcriptase (Invitrogen), according to the manufacturer’s instructions. Quantitative PCRs (qPCRs) were performed on the CFX Connect Real-Time PCR Detection System (Bio-Rad), using iTaq Universal SYBR Green Supermix (Bio-Rad). GAPDH gene was used as reference for data normalization and relative gene expression was measured with the 2^−ΔΔ^*C*_t_ method. qPCR oligo pairs for *PAK1* gene: 5′-CCTGCACCGAAACCGAGTTA-3′ (Fwd) and 5′-TAGGAGTCCCACACAGGGTC-3′ (Rev).

### RNA-sequencing data analysis

RNA isolated using RNeasy Plus Kit (Qiagen) was used for RNA-Sequencing analysis. Library preparation, RNA-Sequencing analysis and reads quality control was performed by Genewiz Company (Germany). Gene set enrichment analysis (GSEA) and Gene Ontology (GO) were performed on normalized data to pinpoint specific gene signatures underpinning resistance to ET ad CDK4/6i.

### Western blot

Cells were lysed with RIPA lysis buffer (sc-24948, Santa Cruz, USA) according to the protocol supplied. Tumor xenograft samples were lysated in RIPA lysis buffer as well, supplemented with protease inhibitors through TissueLyser LT (Qiagen) according to manufacture instructions. Whole cell lysates (30 µg) were separated by SDS-PAGE, transferred to nitrocellulose through Trans-Blot® Turbo™ RTA Mini Nitrocellulose Transfer Kit (Biorad). Membranes were subjected to immunoblot analyses using primary antibodies against phosphorylated RB (Ser780) #8180 1:1000 (Cell signaling technology), phosphorylated RB (Ser807/811) #8516 1:1000 (Cell signaling technology), RB (IF-8) #sc102 1:1000 (santa cruz biotechnology), Er-α (F-10) #sc-8002 1:200 (santa cruz biotechnology), phosphorylated Fak (Tyr925) sc101680 1:1000 (santa cruz biotechnology), Fak (12GA) #sc56901 1:1000 (santa cruz biotechnology), phosphorylated Src (Tyr416) #2101 1:1000 (Cell signaling technology), c-Src (SRC2) #sc-18 1:1000 (santa cruz biotechnology), Phospho-p44/42 MAPK (Erk1/2) (Thr202/Tyr204) #9101 1:1000 (Cell signalling technology), ERK2 #sc1647 1:1000 (santa cruz biotechnology), β-actin (13E5) #4970 (Cell signalling technology), Phospho-Pak1 (Ser199/Ser204) #09-258 1:1000 (Millipore), Phospho-Pak1/Pak2 (Ser144/Ser141) #2606S (Cell signaling), #09-258 1:1000 (Millipore) Pak1 #2602 1:1000 (Cell signalling technology), GAPDH #sc-32233 1:10000 (santa cruz biotechnology), Phospho-MEK1 (Ser298) #9128 1:1000 (Cell signalling), MEK1/2 (L38C12) #4694 1:1000 (Cell signalling technology). HRP-conjugated anti-rabbit and anti-mouse were used as secondary antibodies (Biorad). Immunoreactive proteins were visualized by enhanced chemiluminescence using SuperSignal™ West Pico PLUS Chemiluminescent Substrate (Thermofisher scientific). Membranes were cut horizontally to probe with multiple antibodies. Films were imaged using Brother MFCL2710DW (Brother) at 300 dpi. All blots derive from the same experiment and they were processed in parallel. Uncropped and unprocessed scans of blots are available in Supplementary materials.

### Xenograft studies

All animal procedures were performed in accordance with institutional guidelines of the University of Naples Animal Care Committee. The 6-week-old female Balb/c (nu + /nu + ) mice (ENVIGO) were housed 5 mice per cage in an on-site housing facility access to standard food and water, in a sterile housing in separate room. MCF7-PAK1 cells (7 × 10^6^ cells per mice) re-suspended in 200 μl of PBS/Matrigel matrix basement membrane (1:1) (Corning #356234) were injected subcutaneously into the right flank of each mouse in accordance with the Declaration of Helsinki. 4 weeks after tumor cell injection, once tumors reached a volume ≥200 mm^3^, mice were randomly assigned (5 *per* group) to receive: 1) vehicle; 2) fulvestrant (5 mg *per* week; subcutaneous injection, s.c.) plus abemaciclib (20 mg/kg/day *via* oral gavage, p.o.), 3) PF-3758309 (15 mg/kg/day *via* oral gavage, p.o.), 4) fulvestrant+abemaciclib + PF-3758309; for 15 days. Animal weights and tumor diameters (with calipers) were measured twice weekly and tumor volume in mm^3^ was calculated with the formula: volume = width2 x length/2. Mice were treated for 2 weeks, 4 h after the last dose of PF-3758309 ± fulvestrant and abemaciclib, mice were euthanized by CO2 and tumor were harvested and snap-frozen in liquid nitrogen before proceeding to total protein exraction as described above.

### Immunohistochemistry staining

To perform the immunostaining for P-Erk (Thr202/Tyr204) and Ki67, 4/5-micron tissue sections were taken from each tumor block. Following 1 h in a hot air oven at 60 °C, slides were loaded on the BenchMark Ultra autostainer (Ventana Medical Systems, Tucson, AZ). The rabbit monoclonal antibody anti- Ki67 (Clone 30-9 - Ventana Medical Systems, Tucson, AZ) was used according to a standard Assay protocol. The monoclonal mouse anti P-Erk (Santa Cruz Biotechnology, Dallas, USA) was manually dispensed during the antibody tritation step using a 1:500 diluition in Antibody diluent (Ventana Medical Systems, Tucson, AZ) after an antigen unmasking performed by a standard automated method (64 min Utra CC1). The optimized incubation time was chosen after different test on murine tissue in 36 min. Then, slides were counterstained with hematoxylin and bluing reagents. Post-run, the stained slides were sequentially dehydrated with graded alcohols 70%, 95% and 100%, then rinsed in xylene and mounted with Micromout medium (Diapath, Italy).

### Reporting summary

Further information on research design is available in the Nature Research Reporting Summary linked to this article.

## Supplementary information

Supplementary materials

Reporting Summary

## Data Availability

The RNA-seq data that support this study have been deposited into National Center for Biotechnology Information Gene Expression Omnibus (GEO) DataSets using the accession number GSE227102. All other relevant data are included in the manuscript and its Supplementary materials file or available from the corresponding author upon reasonable request.
