# Supplementary material for: Pak1 pathway hyper-activation mediates resistance to endocrine therapy and CDK4/6 inhibitors in ER+ breast cancer
Source: NPJ Breast Cancer. 2023 May 31;9:48. doi: 10.1038/s41523-023-00556-9 (PMC10232479; doi:10.1038/s41523-023-00556-9)

Supplementary Figure 1. Sensitivity of MCF-7-FAR and T47D-FAR cells to single drugs

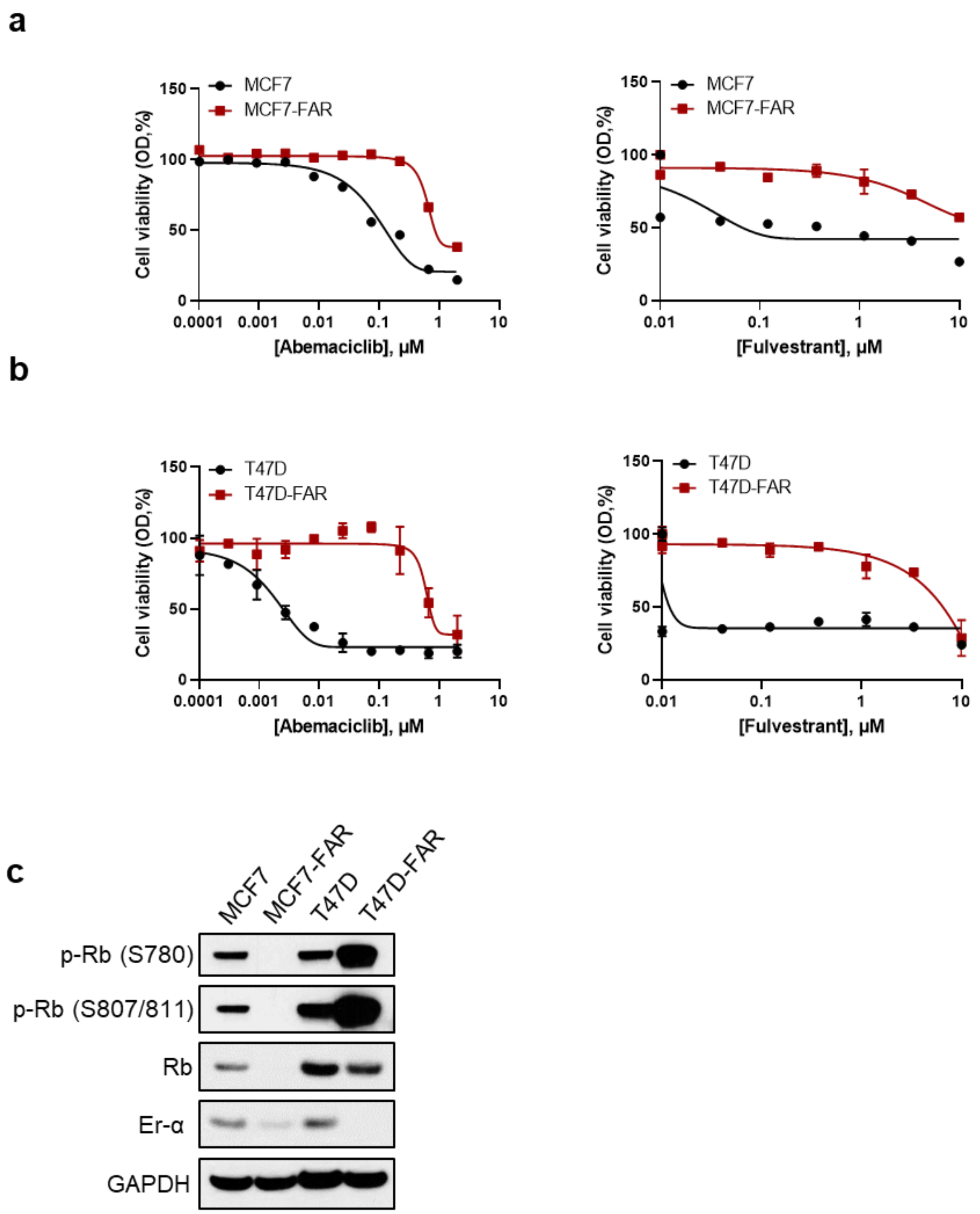

Supplementary Figure 1. Dose-response curves of MCF7, MCF7-FAR (**a**), T47D, T47D-FAR (**b**) exposed to increasing doses of fulvestrant or abemaciclib up to 10 µM or 2.5 µM concentrations, respectively, every 72 hours for 1 week, and then stained with crystal violet solution. Each data point represents the percent of cell viability relative to vehicle-treated controls, and shown as mean ± SD from three independent experiments. Western blot for p-Rb (S780), p-Rb (S807/811), Rb, Er-α in MCF7, T47D parental and –FAR cells, treated with fulvestrant (1µM) and abemaciclib (0.25µM) for 18 hours. GAPDH was used as a loading control. Images are representatives from three independent experiments (**c**).

Supplementary Figure 2. Analysis of cytoskeleton rearrangement and migratory capability of FAR cells

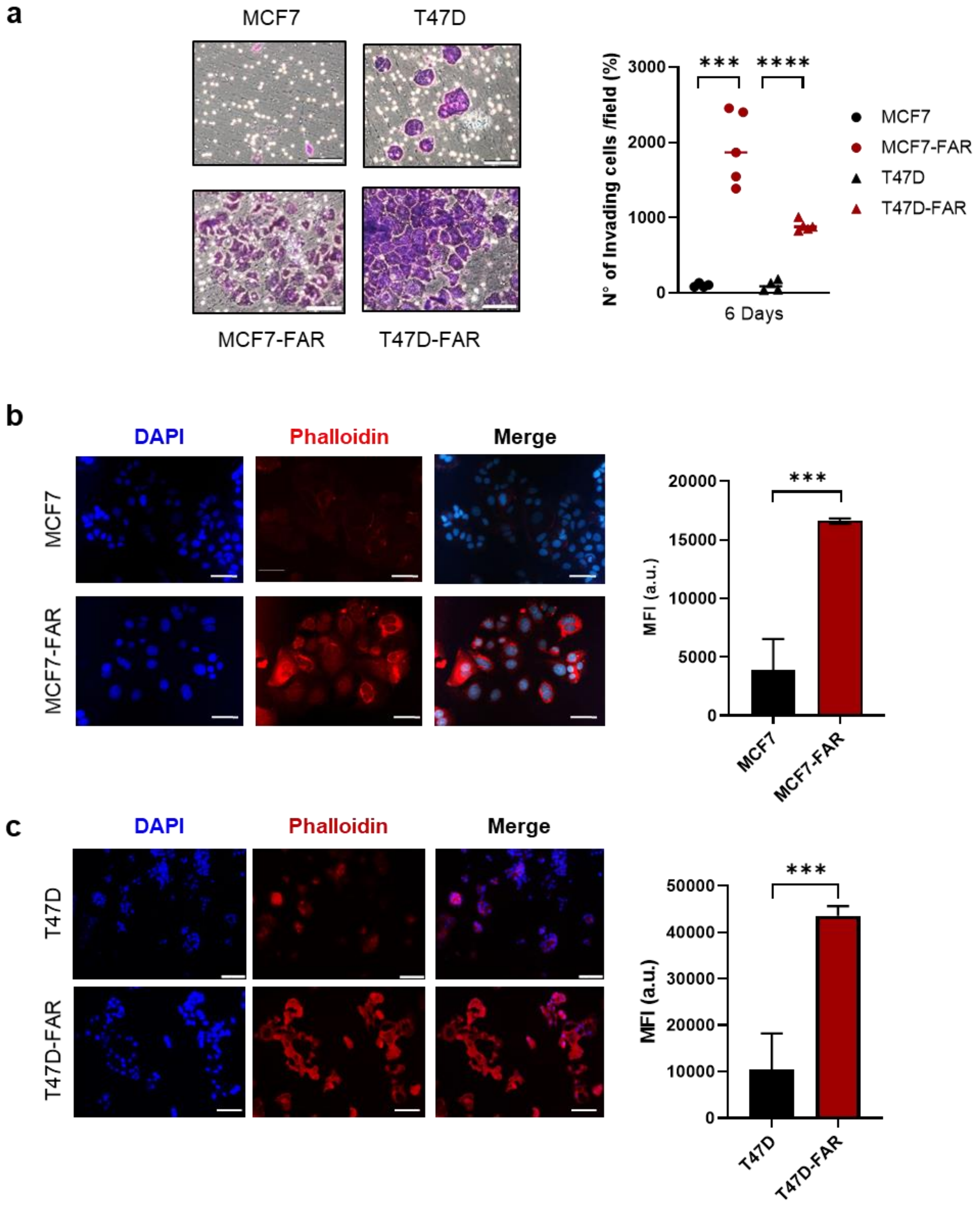

Supplementary Figure 2. Representative images of crystal violet stained MCF7, MCF7-FAR, T47D, or T47D-FAR invading cells through matrigel-coated transwell. All images were captured at 20x magnification (Bars = 200 µm) (**a**). Scatter plot showing the mean of cell number *per* field reported as percentage relative to parental cells (**a, right**). Representative immunofluorescence (IF) staining for DAPI (blu) and Phalloidin (red) and merged images of MCF7 and MCF7-FAR cells (**b**) or T47D and T47D-FAR cells (**c**). All images were capture at 40x magnification (Bars = 50 µm). Quantification of mean of fluorescence intensities was analyzed by ImageJ software. Data are plotted in the bar graphs as mean ± SD of MCF7-FAR relative to MCF7 (**b, right**), and of T47D-FAR relative to T47D (**c, right**). For all panels, images are representatives from three independent experiments, performed in triplicates (****p*<0.001, **** p<0.0001; *Student's T-test*).

Supplementary Figure 3. Analysis of protein expression levels of Pak1 in parental and FAR MCF7 or T47D.

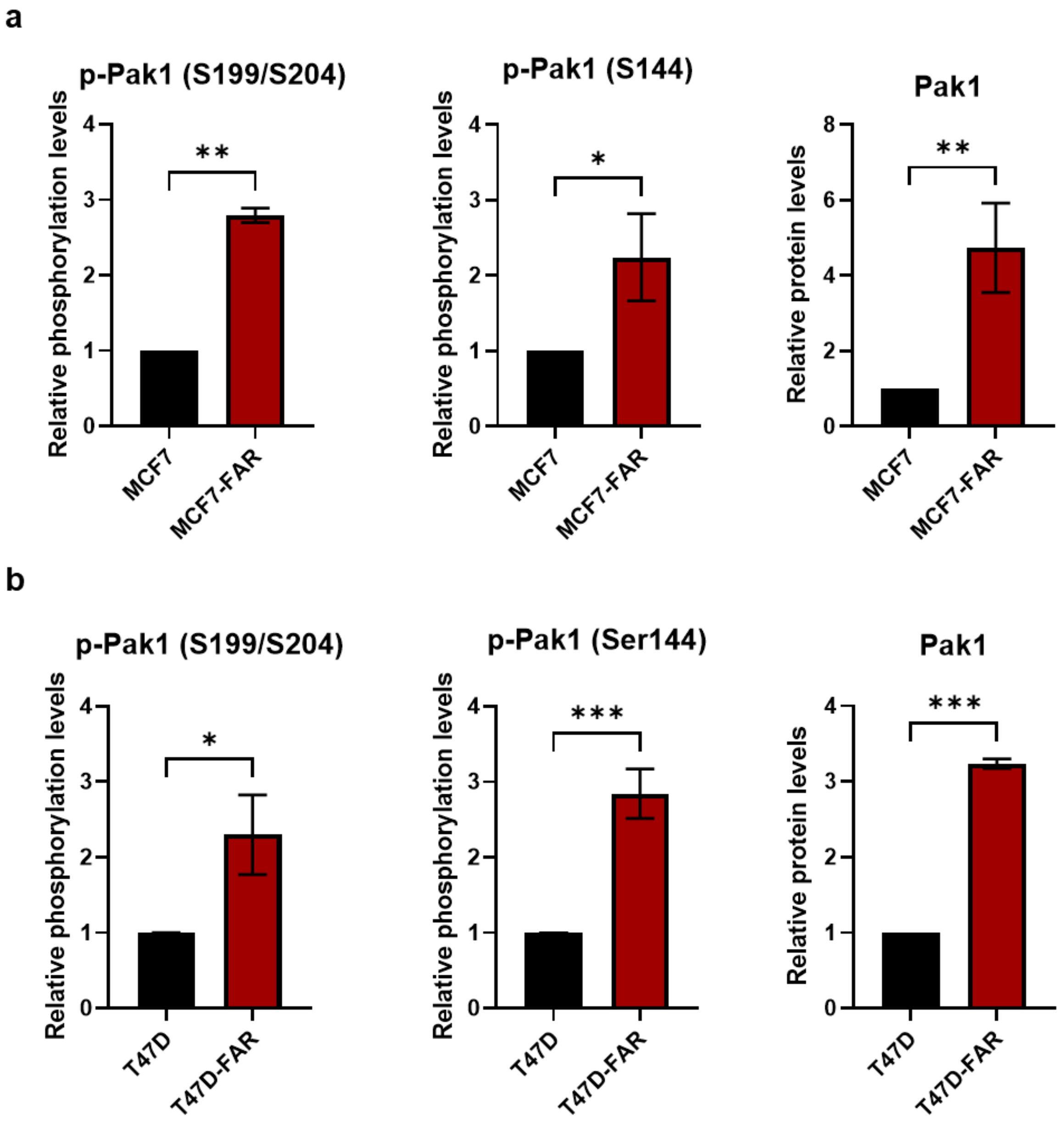

Supplementary Figure 3. Whole total lysates prepared from MCF7 and MCF7-FAR (**a**) or T47D, and T47D-FAR (**b**) were subjected to western blot analysis for p-Pak1 (S199/S204), p-Pak1 (S144); Pak1. Densitometric quantitation of the resulting molecular species performed using data from three independent experiments is reported in bar charts. Data are expressed as mean ± standard deviation (**p<0,05;* ***p*<0.01, *** p<0.001; *Student's T-test*).

Supplementary Figure 4: Invasive capability of *PAK1* over-expressing and FAR cells.

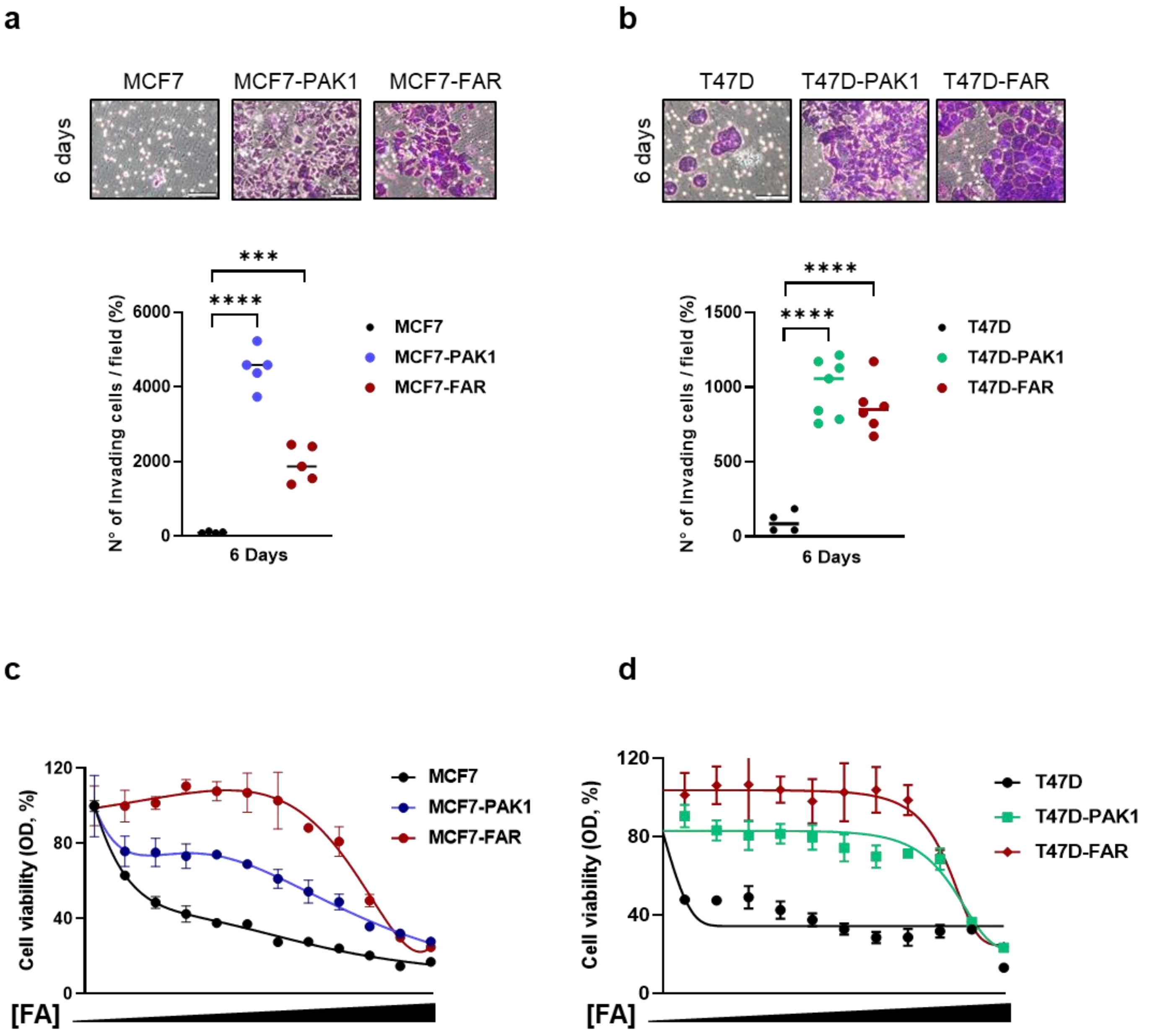

Supplementary Figure 4. Representative images of crystal violet stained MCF7, MCF7-PAK1, and MCF7-FAR (**a, top**) or T47D, T47D-PAK1, and T47D-FAR (**b, top**) invading cells through matrigel-coated transwell. All images were capture at 20x magnification (Bars = 200 µm). Images are representative from three independent experiments, performed in triplicates. Scatter plot showing the mean of cell number *per* field reported as percentage relative to parental cells for MCF7 (**a, bottom**) and for T47D (**b, bottom**) models. Dose-response curves of MCF7, MCF7-PAK1, and MCF7-FAR (**c**) T47D, T47D-PAK1, and T47D-FAR (**d**) exposed to increasing doses of fulvestrant and abemaciclib (FA) up to 10 µM or 2.5 µM concentrations, respectively, every 72 hours for 1 week, and then stained with crystal violet solution. Each data point represents the percent of cell viability relative to vehicle-treated controls, and shown as mean ± SD from two independent experiments. For all panels, (****p*<0.001, **** p<0.0001; *Student's T-test*).

Supplementary Figure 5. *PAK1* down-modulation impairs invasive capacity of MCF7-FAR and T47D-FAR cells

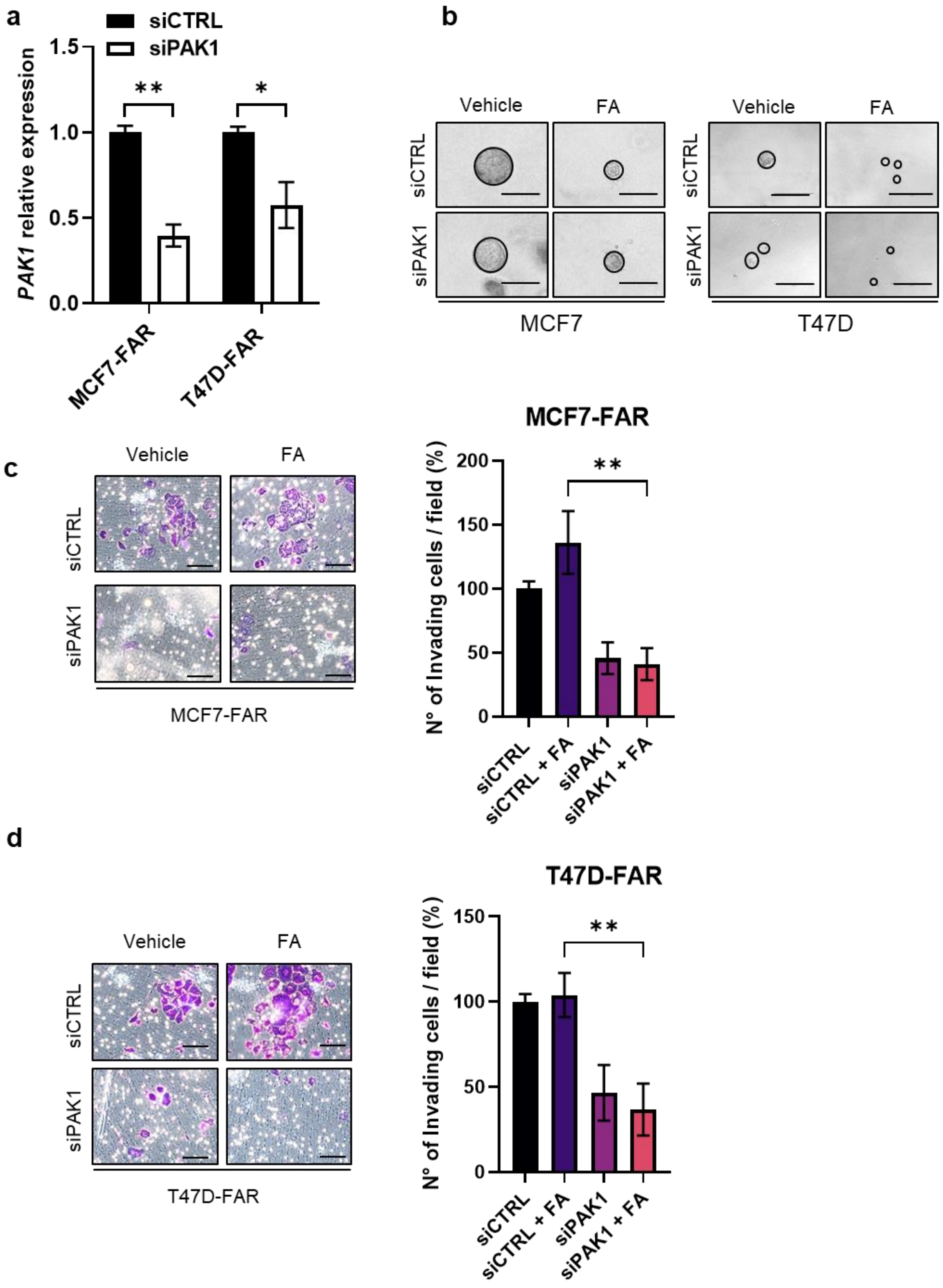

Supplementary Figure 5. Quantitative-PCR for *PAK1* gene expression 24 hours after siRNAs transfection in MCF7-FAR and T47D-FAR cells. Data are expressed as mean ± SD relative to cells transfected with siRNA scrambled (siCTRL), from three independent experiments performed in triplicates (**p*< 0.05; ***p*< 0.01, *Student's T-test*) (**a**). Representative images of spheroids embedded in collagen type I matrix set up from MCF7 (**b, left**) or T47D cells (**b, right**) transfected with 40 nM siRNA scrambled (siCTRL) or siRNA targeting *PAK1* (siPAK1) in presence of vehicle or 1μM fulvestrant and 0.25 μM abemaciclib for 6 days. All images were capture at 20x magnification (Bars = 200 μm). Images are representative from three independent experiments performed in triplicate. Representative images of crystal violet stained MCF7-FAR (c, left) or T47D-FAR (d, right) invading cells through matrigel-coated transwell, treated as described in panel b. Images are representative from three independent experiments, performed in triplicate. Bar graphs showing the mean of cell number *per* field reported as percentage relative to vehicle-treated controls for MCF7-FAR (c, right) and for T47D-FAR (d, right). For all panels, (**p<0.01, Student's T-test).

Supplementary Figure 6. Impairment of FAR cell growth and invasion upon NVS-PAK1-1 treatment

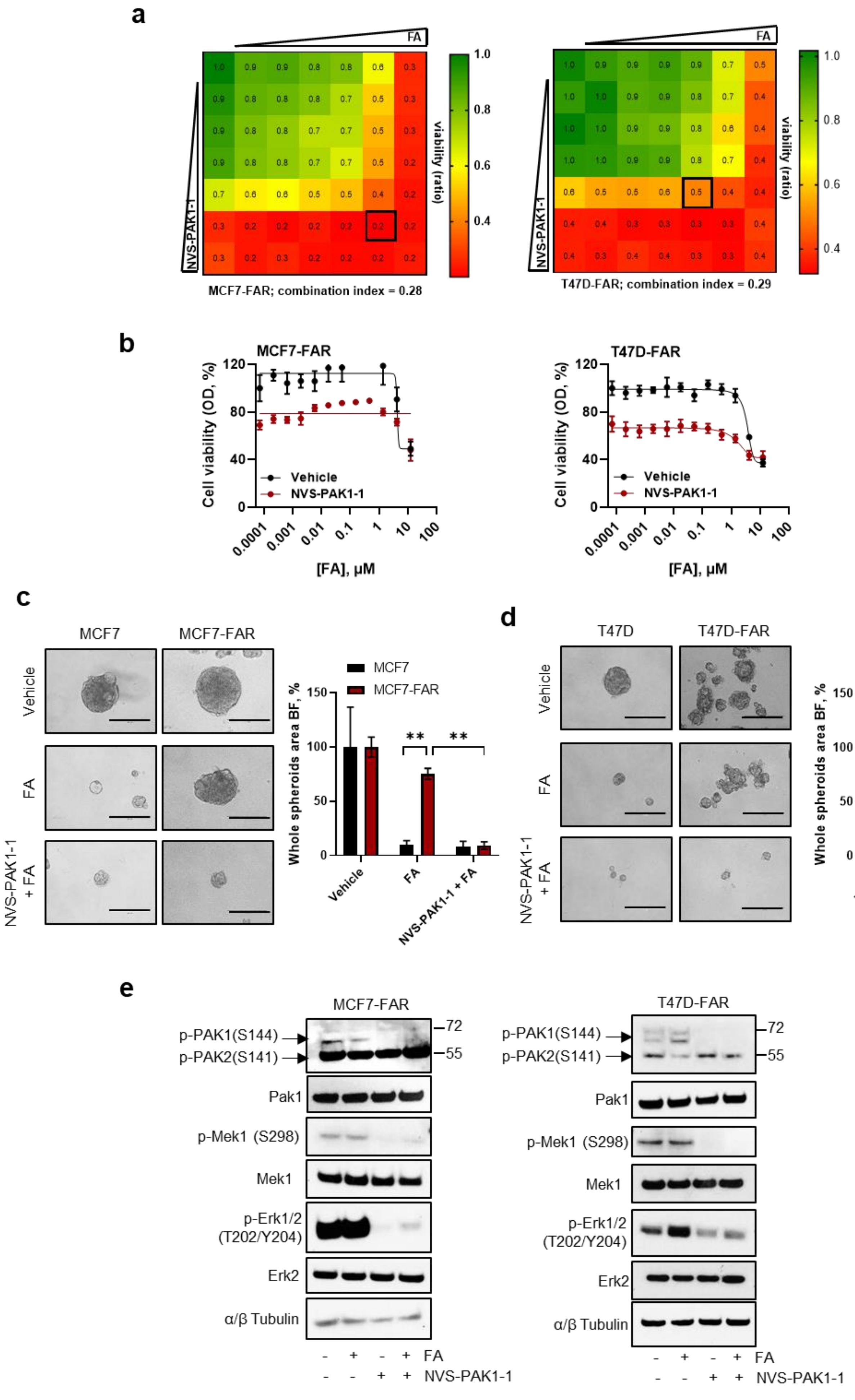

Supplementary Figure 6. Viability assays to test synergy between fulvestrant and abemaciclib (FA) and NVS-PAK1-1. Cells were treated with increasing concentrations of FA and NVS-PAK1-1 (up to 10μM and 100μM, respectively) alone or in combination every 72 h until vehicle-treated controls reached ~90% of confluence. Intensity values of cell monolayers stained with crystal violet were used to perform the Chou-Talalay test. Numbers inside each box indicate the *ratio* of viable treated cells to untreated cells from three independent experiments for MCF7-FAR (**a, left**) and for T47D-FAR (**a, right**). Dose-response curves of MCF7-FAR (**b**, **left**) or T47D-FAR (**b**, **right**) exposed to increasing doses of fulvestrant and abemaciclib (FA, up to 10μM fulvestrant and 2.5 μM abemaciclib) in presence or not of 10μM NVS-PAK-1 for 1 week. Representative images of MCF7 and MCF7-FAR (**c**, **left**) or T47D and T47D-FAR (**d**, **left**) spheroids exposed to 400nM fulvestrant and 100nM abemaciclib (FA) ± 5 μM NVS-PAK1-1. Bar graphs showing percentage of whole spheroids area upon fulvestrant and abemaciclib (FA) ± NVS-PAK1-1 treatment compared to spheroids treated with vehicle (plotted as 100%) are reported for MCF7 (**c, right**) and T47D (**d, right**) models. Western Blot analysis of MCF7-FAR (**e, left**) and T47D-FAR (**e, right**) treated with fulvestrant and abemaciclib (FA, 1μM and 0.25 μM, respectively), NVS-PAK1-1 (40μM) or the combination for 48 hours. α/β tubulin was used as loading control. For all panels, data are plotted as means ± SD of three independent experiments performed in triplicate or quadruplicate (**$p<$ 0.01; ****$p<$ 0.0001; 2way ANOVA Bonferroni's multiple comparisons).

Supplementary Fig. 7: PF309 and NVS-PAK1-1 affect invasion of *PAK1* over-expressing and FAR cells

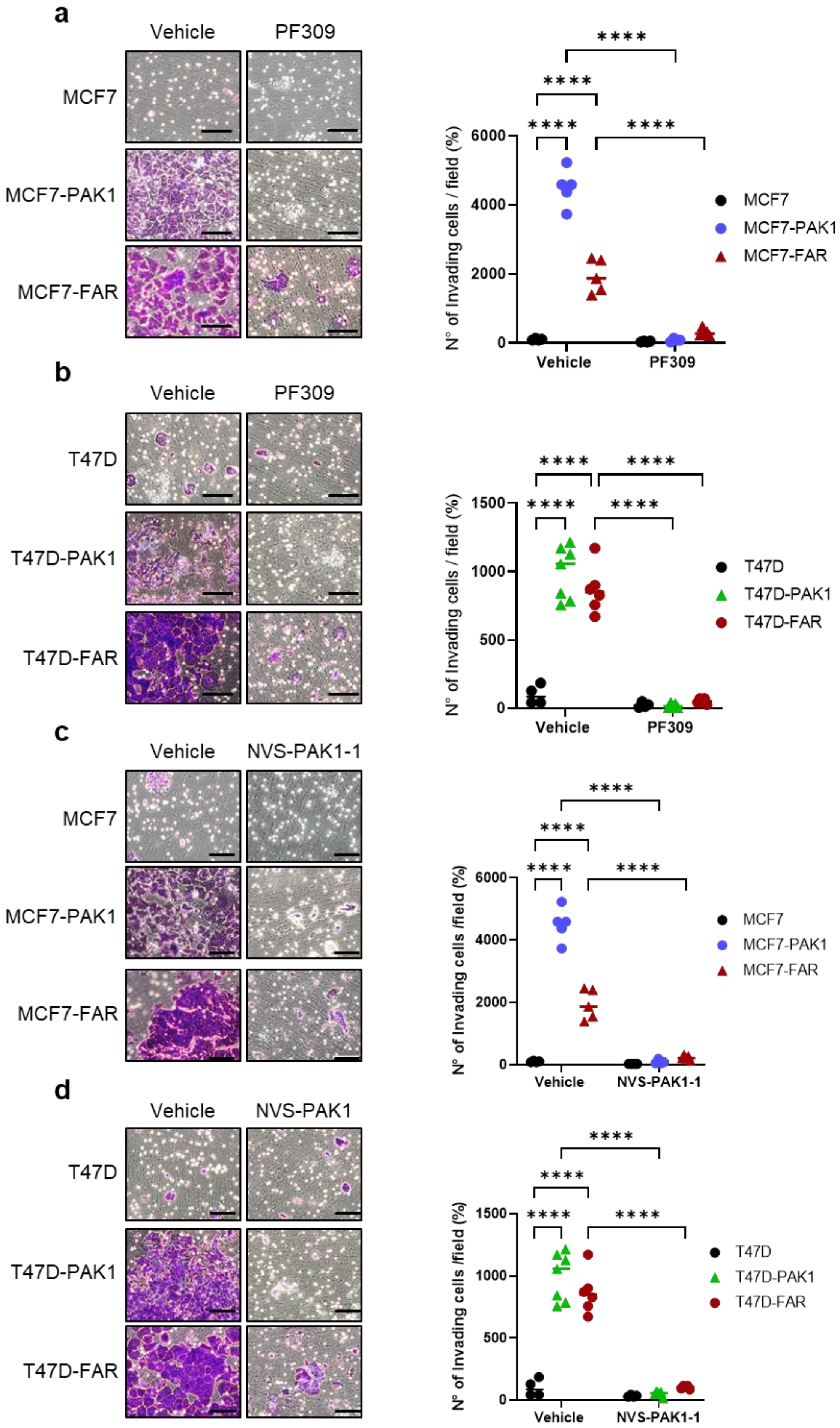

Supplementary Figure 7. Representative images of crystal violet stained MCF7, MCF7-PAK1, and MCF7-FAR invading cells, through matrigel-coated transwell, treated with 10nM PF309 (**a, left**) or NVS-PAK1-1 10μM (**c, left**) and T47D, T47D-PAK1 and T47D-FAR cells treated with 10nM PF309 (**b, left**) or NVS-PAK1-1 10 μM (**d, left**) for 6 days. All images were captured at 20x magnification (Bars = 200 μm). Images are representative from three independent experiments, performed in triplicates. Scatter plots showing the mean of cell number *per* field reported as percentage relative to parental cells MCF7 or T47D (**a-d, right**). Middle lines represent median. For all panels (***p<0.001, **** p<0.0001; 2way ANOVA Bonferroni's multiple comparisons).

Supplementary Figure 8. Body weight of Balb/c female

a

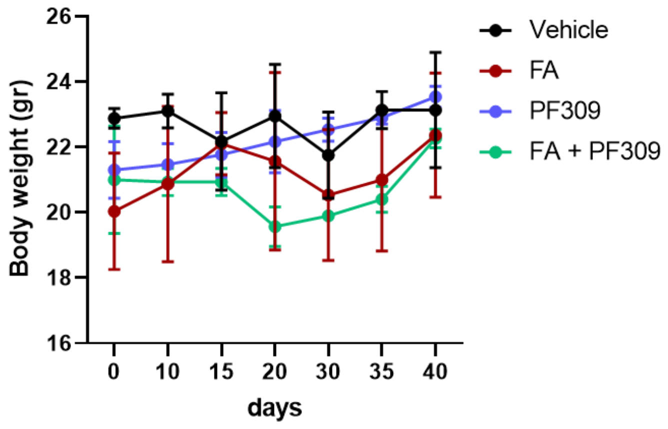

Supplementary Figure 8. Line chart showing body weight trend of Balb/c nude mice treated with vehicle, fulvestrant and abemaciclib (FA), PF309 and their combination as described in Fig.6 (**a**).

Figure 2

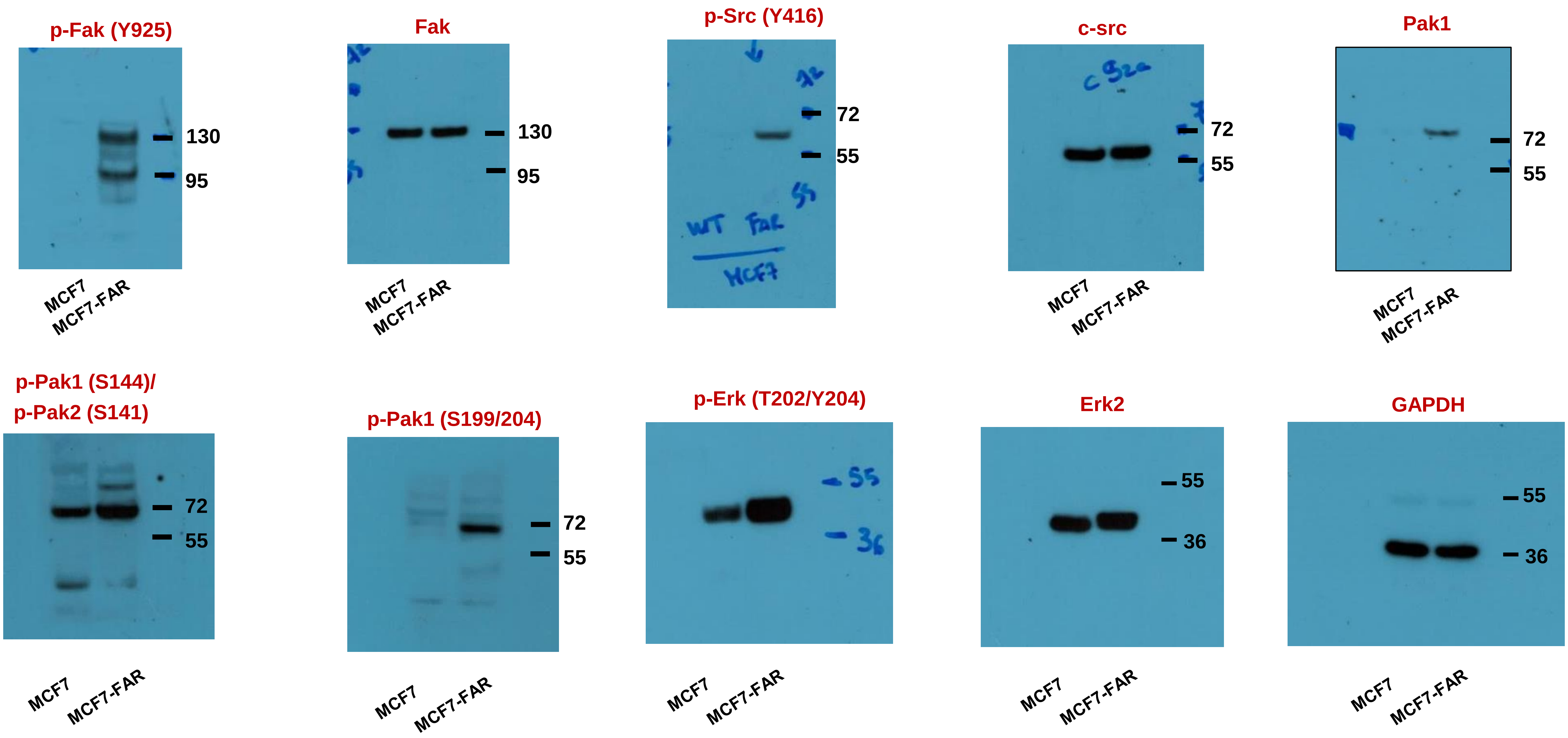
p-Fak (Y925)
130
95
MCF7
MCF7-FAR
Fak
130
95
MCF7
MCF7-FAR
p-Src (Y416)
72
55
WT FAR
MCF7
c-src
72
55
MCF7
MCF7-FAR
Pak1
72
55
MCF7
MCF7-FAR
p-Pak1 (S144)/
p-Pak2 (S141)
72
55
MCF7
MCF7-FAR
p-Pak1 (S199/204)
72
55
MCF7
MCF7-FAR
p-Erk (T202/Y204)
55
36
MCF7
MCF7-FAR
Erk2
55
36
MCF7
MCF7-FAR
GAPDH
55
36
MCF7
MCF7-FAR

Figure 2

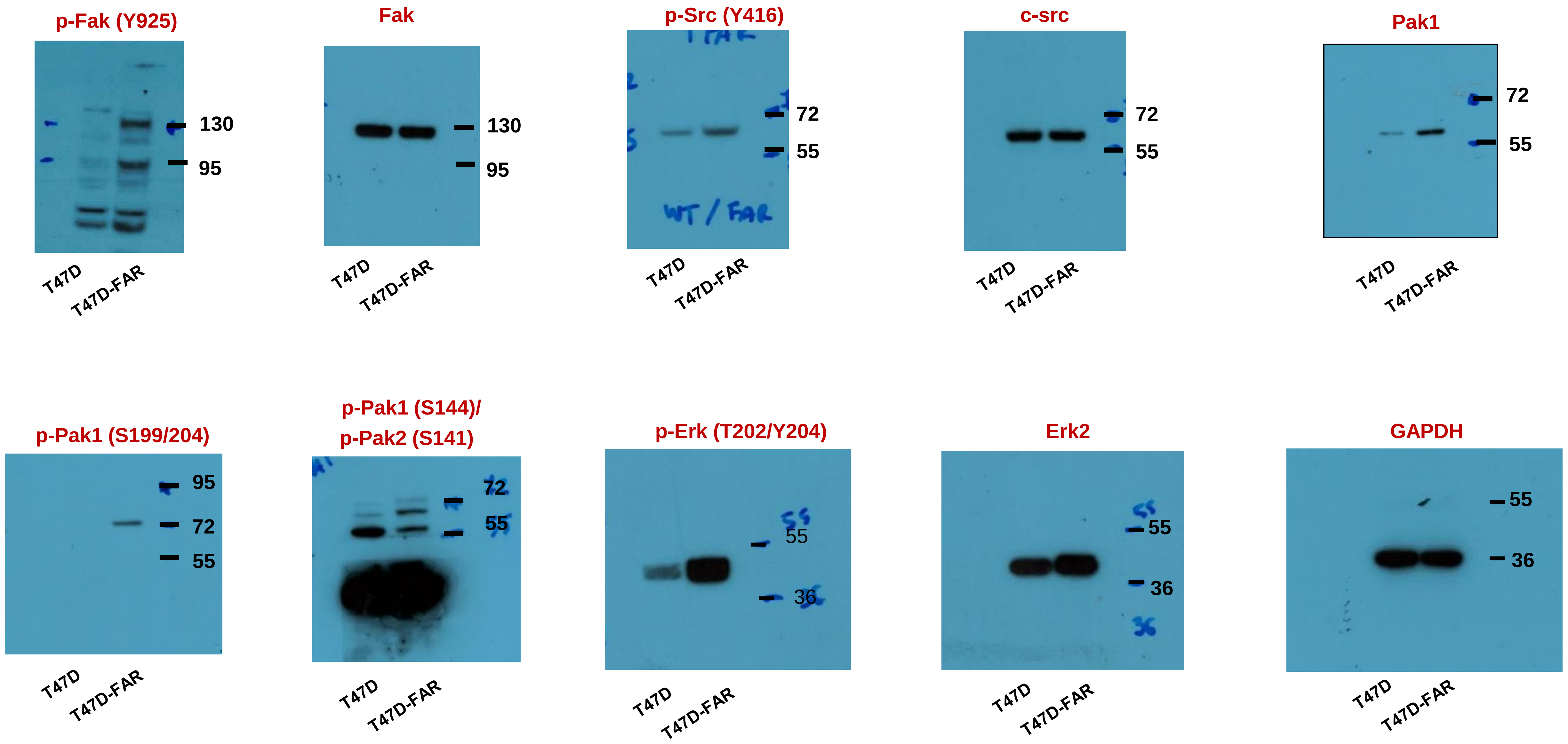
p-Fak (Y925)
130
95
T47D
T47D-FAR
Fak
130
95
T47D
T47D-FAR
p-Src (Y416)
72
55
T47D
T47D-FAR
c-src
72
55
T47D
T47D-FAR
Pak1
72
55
T47D
T47D-FAR
p-Pak1 (S199/204)
95
72
55
T47D
T47D-FAR
p-Pak1 (S144)/
p-Pak2 (S141)
72
55
T47D
T47D-FAR
p-Erk (T202/Y204)
55
36
T47D
T47D-FAR
Erk2
55
36
T47D
T47D-FAR
GAPDH
55
36
T47D
T47D-FAR

Figure 3

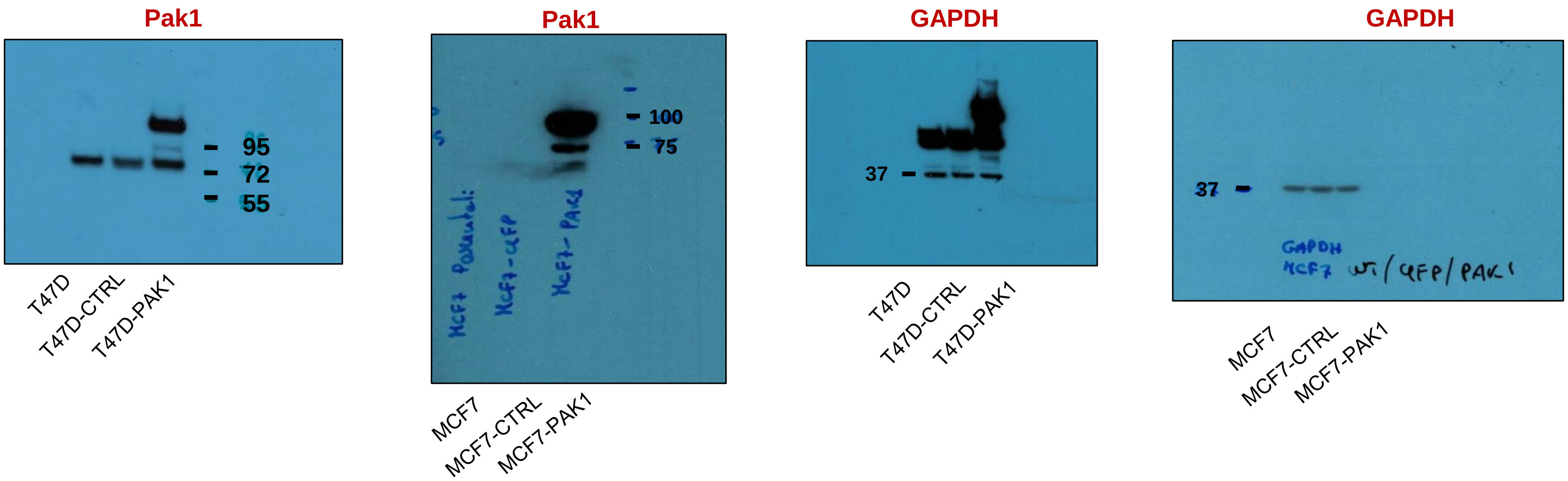
Pak1
95
72
55
T47D
T47D-CTRL
T47D-PAK1
Pak1
100
75
MCF7
MCF7-CTRL
MCF7-PAK1
GAPDH
37
T47D
T47D-CTRL
T47D-PAK1
GAPDH
37
MCF7
MCF7-CTRL
MCF7-PAK1

Figure 4

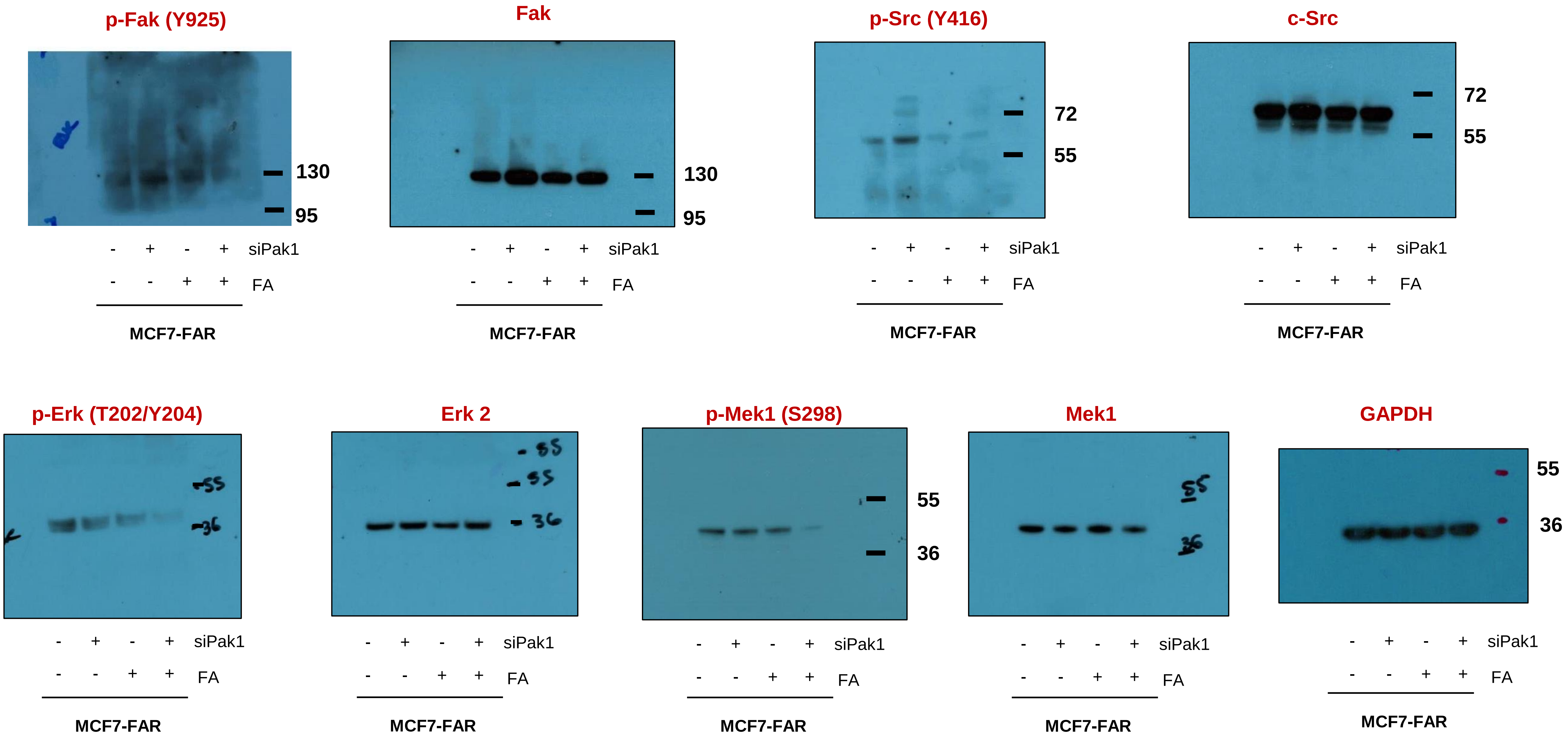
p-Fak (Y925)
130
95
- + - + siPak1
- - + + FA
MCF7-FAR
Fak
130
95
- + - + siPak1
- - + + FA
MCF7-FAR
p-Src (Y416)
72
55
- + - + siPak1
- - + + FA
MCF7-FAR
c-Src
72
55
- + - + siPak1
- - + + FA
MCF7-FAR
p-Erk (T202/Y204)
- + - + siPak1
- - + + FA
MCF7-FAR
Erk 2
- + - + siPak1
- - + + FA
MCF7-FAR
p-Mek1 (S298)
55
36
- + - + siPak1
- - + + FA
MCF7-FAR
Mek1
- + - + siPak1
- - + + FA
MCF7-FAR
GAPDH
55
36
- + - + siPak1
- - + + FA
MCF7-FAR

Figure 4

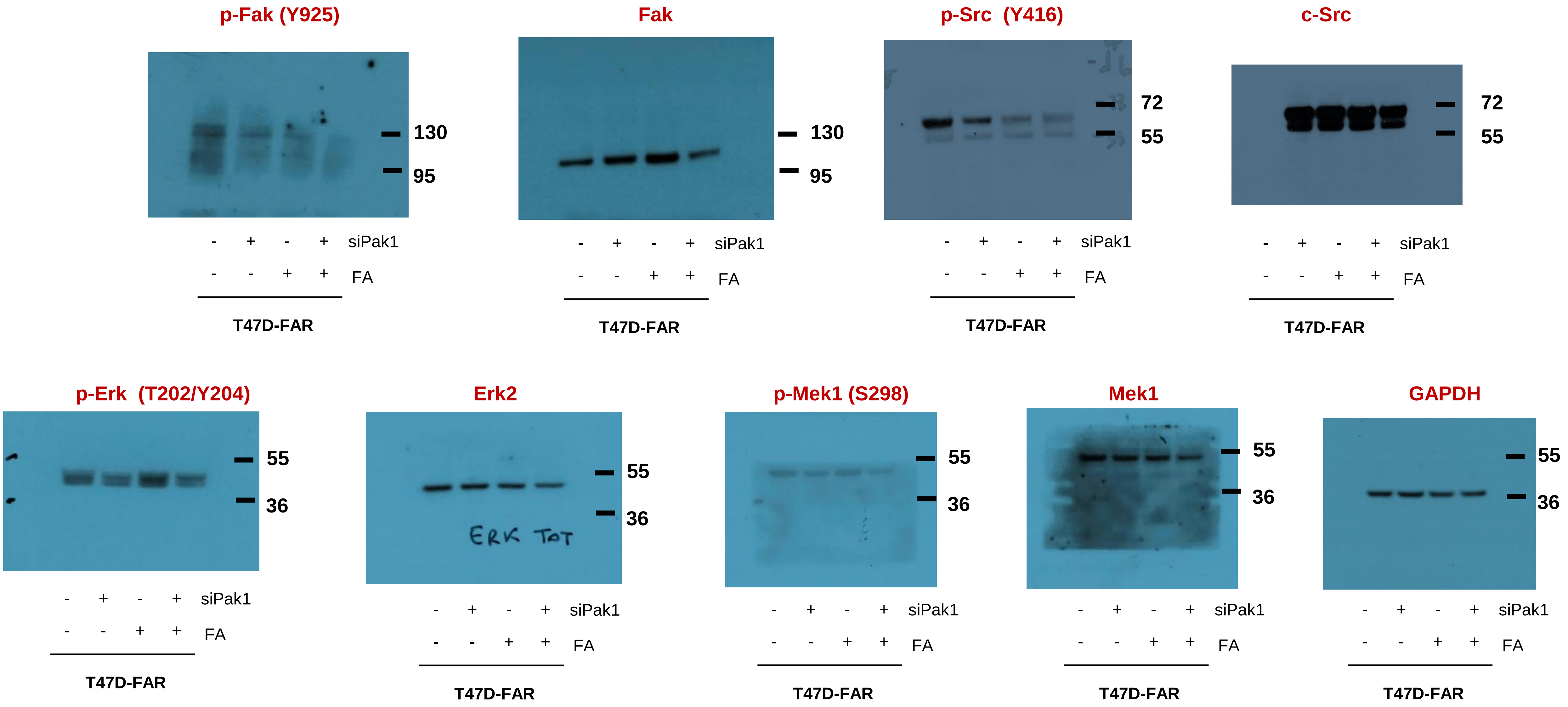

p-Fak (Y925)
130
95
- + - + siPak1
- - + + FA
T47D-FAR
Fak
130
95
- + - + siPak1
- - + + FA
T47D-FAR
p-Src (Y416)
72
55
- + - + siPak1
- - + + FA
T47D-FAR
c-Src
72
55
- + - + siPak1
- - + + FA
T47D-FAR
p-Erk (T202/Y204)
55
36
- + - + siPak1
- - + + FA
T47D-FAR
Erk2
55
36
ERK TOT
- + - + siPak1
- - + + FA
T47D-FAR
p-Mek1 (S298)
55
36
- + - + siPak1
- - + + FA
T47D-FAR
Mek1
55
36
- + - + siPak1
- - + + FA
T47D-FAR
GAPDH
55
36
- + - + siPak1
- - + + FA
T47D-FAR

Figure 5

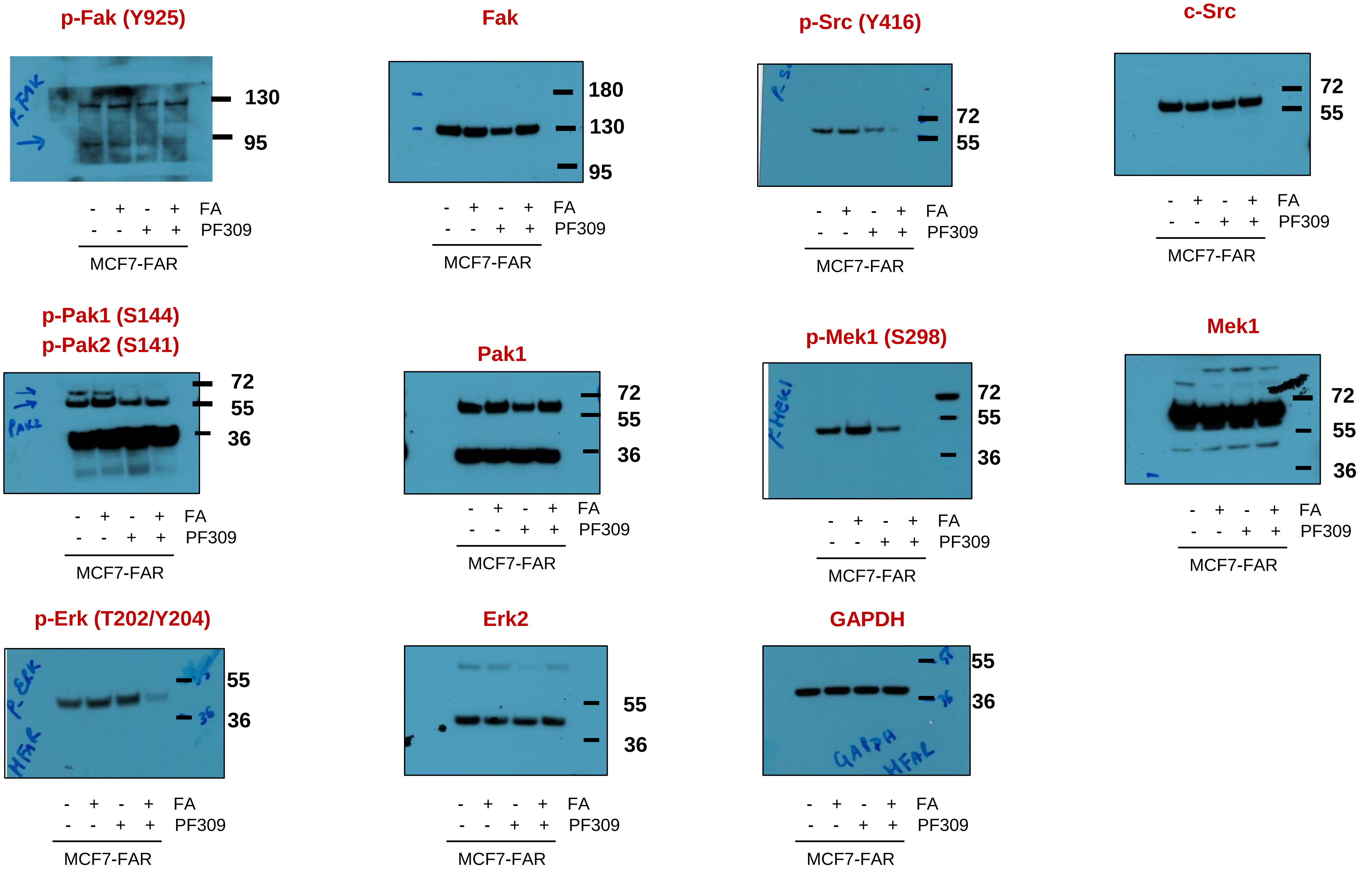

p-Fak (Y925)
130
95
Fak
180
130
95
p-Src (Y416)
72
55
c-Src
72
55
p-Pak1 (S144)
p-Pak2 (S141)
72
55
36
Pak1
72
55
36
p-Mek1 (S298)
72
55
36
Mek1
72
55
36
p-Erk (T202/Y204)
55
36
Erk2
55
36
GAPDH
55
36
- + - + FA
- - + + PF309
MCF7-FAR

Figure 5

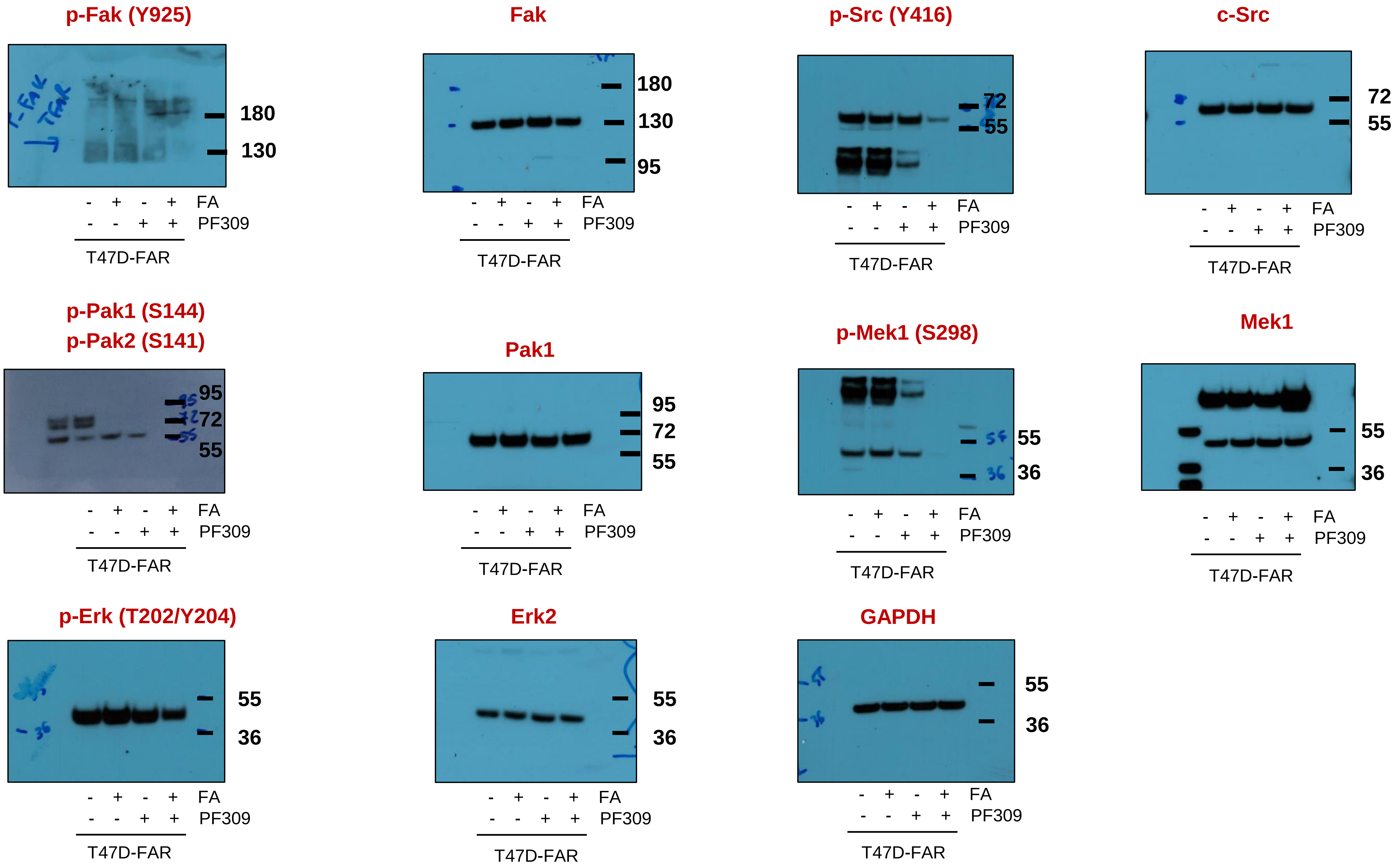

p-Fak (Y925)
180
130
- + - + FA
- - + + PF309
T47D-FAR
Fak
180
130
95
- + - + FA
- - + + PF309
T47D-FAR
p-Src (Y416)
72
55
- + - + FA
- - + + PF309
T47D-FAR
c-Src
72
55
- + - + FA
- - + + PF309
T47D-FAR
p-Pak1 (S144)
p-Pak2 (S141)
95
72
55
- + - + FA
- - + + PF309
T47D-FAR
Pak1
95
72
55
- + - + FA
- - + + PF309
T47D-FAR
p-Mek1 (S298)
55
36
- + - + FA
- - + + PF309
T47D-FAR
Mek1
55
36
- + - + FA
- - + + PF309
T47D-FAR
p-Erk (T202/Y204)
55
36
- + - + FA
- - + + PF309
T47D-FAR
Erk2
55
36
- + - + FA
- - + + PF309
T47D-FAR
GAPDH
55
36
- + - + FA
- - + + PF309
T47D-FAR

Figure 6

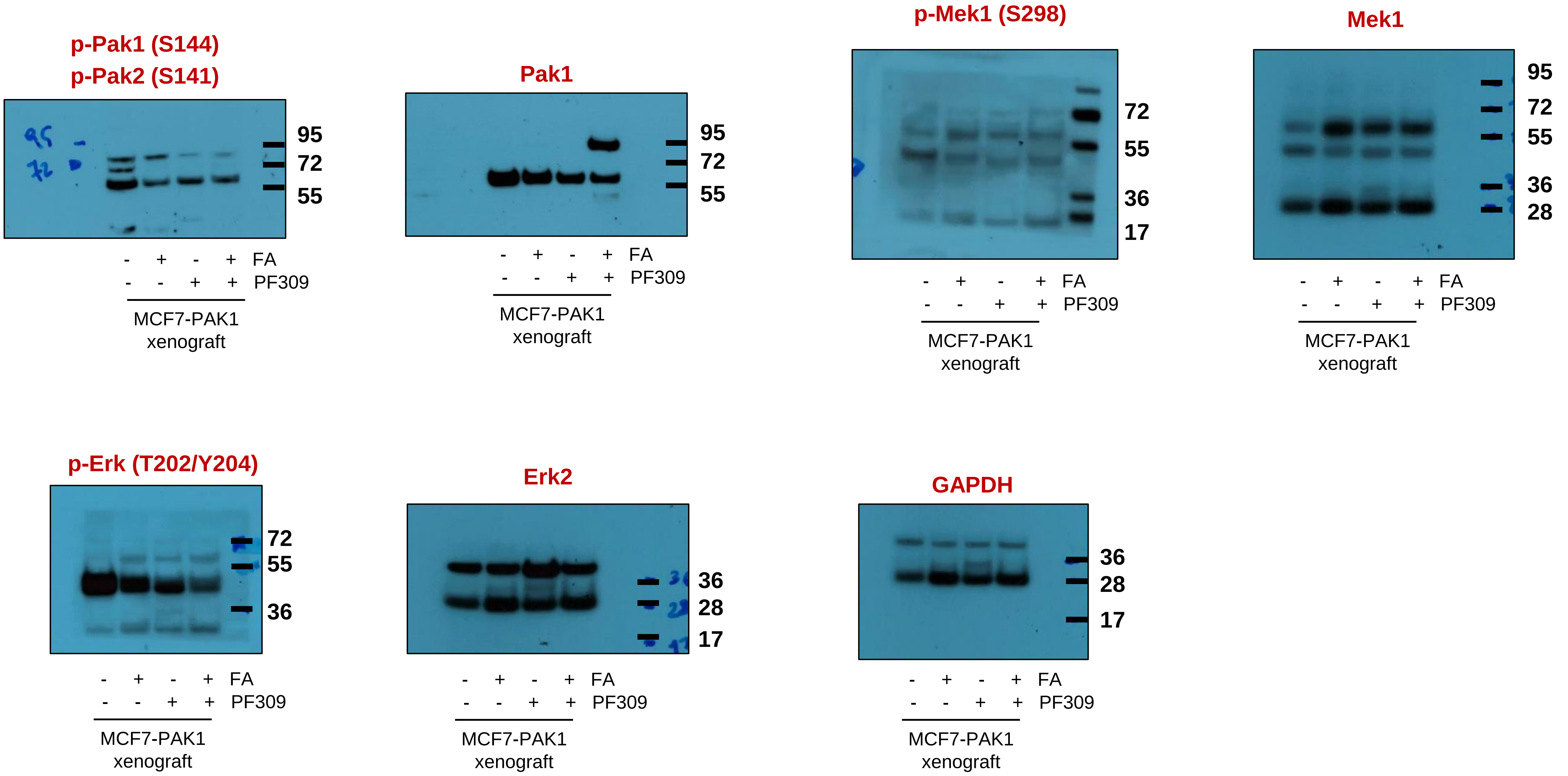
p-Pak1 (S144)
p-Pak2 (S141)
95
72
55
- + - + FA
- - + + PF309
MCF7-PAK1 xenograft
Pak1
95
72
55
- + - + FA
- - + + PF309
MCF7-PAK1 xenograft
p-Mek1 (S298)
72
55
36
17
- + - + FA
- - + + PF309
MCF7-PAK1 xenograft
Mek1
95
72
55
36
28
- + - + FA
- - + + PF309
MCF7-PAK1 xenograft
p-Erk (T202/Y204)
72
55
36
- + - + FA
- - + + PF309
MCF7-PAK1 xenograft
Erk2
36
28
17
- + - + FA
- - + + PF309
MCF7-PAK1 xenograft
GAPDH
36
28
17
- + - + FA
- - + + PF309
MCF7-PAK1 xenograft

Supplementary Figure 1

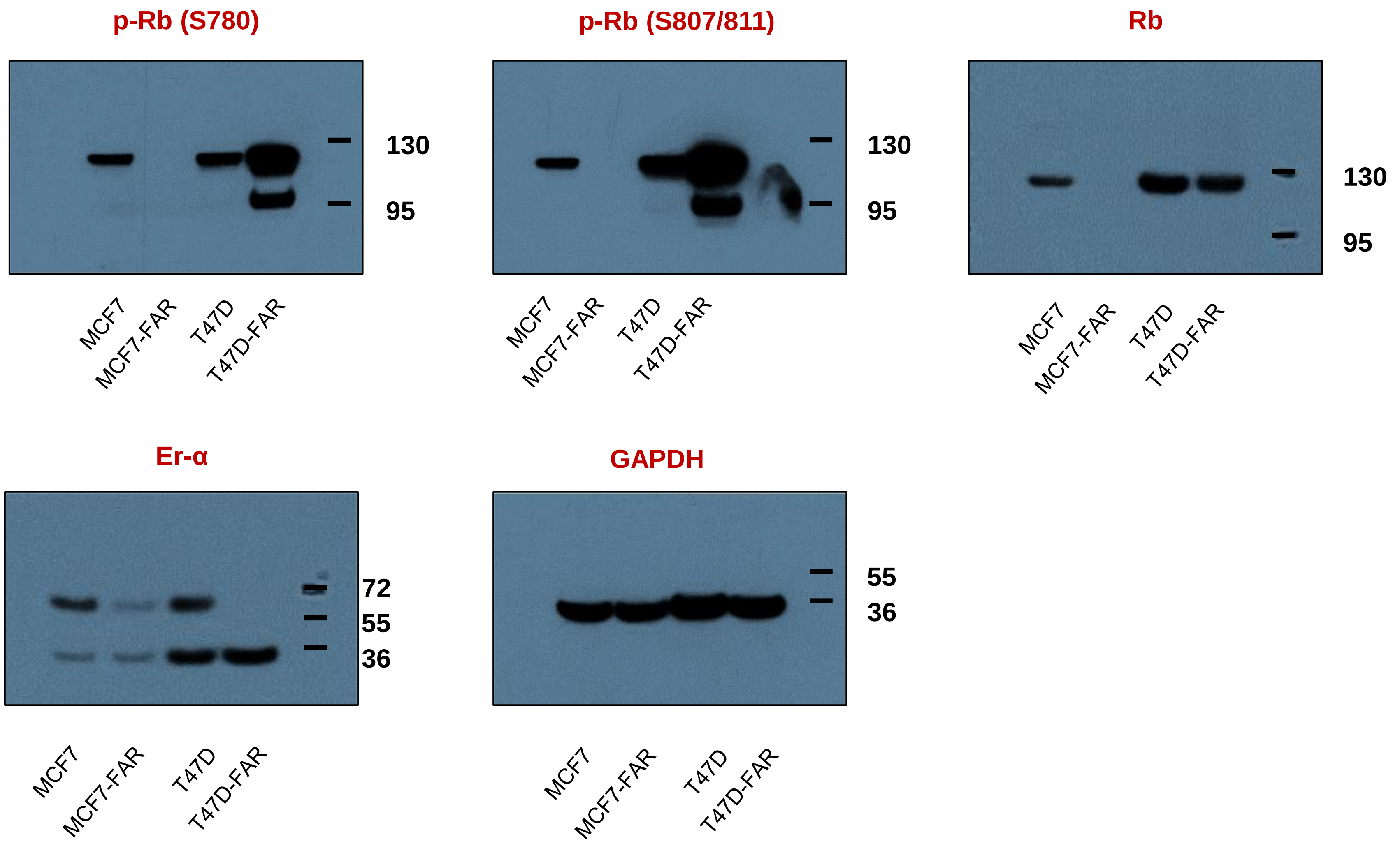

Supplementary Figure 6

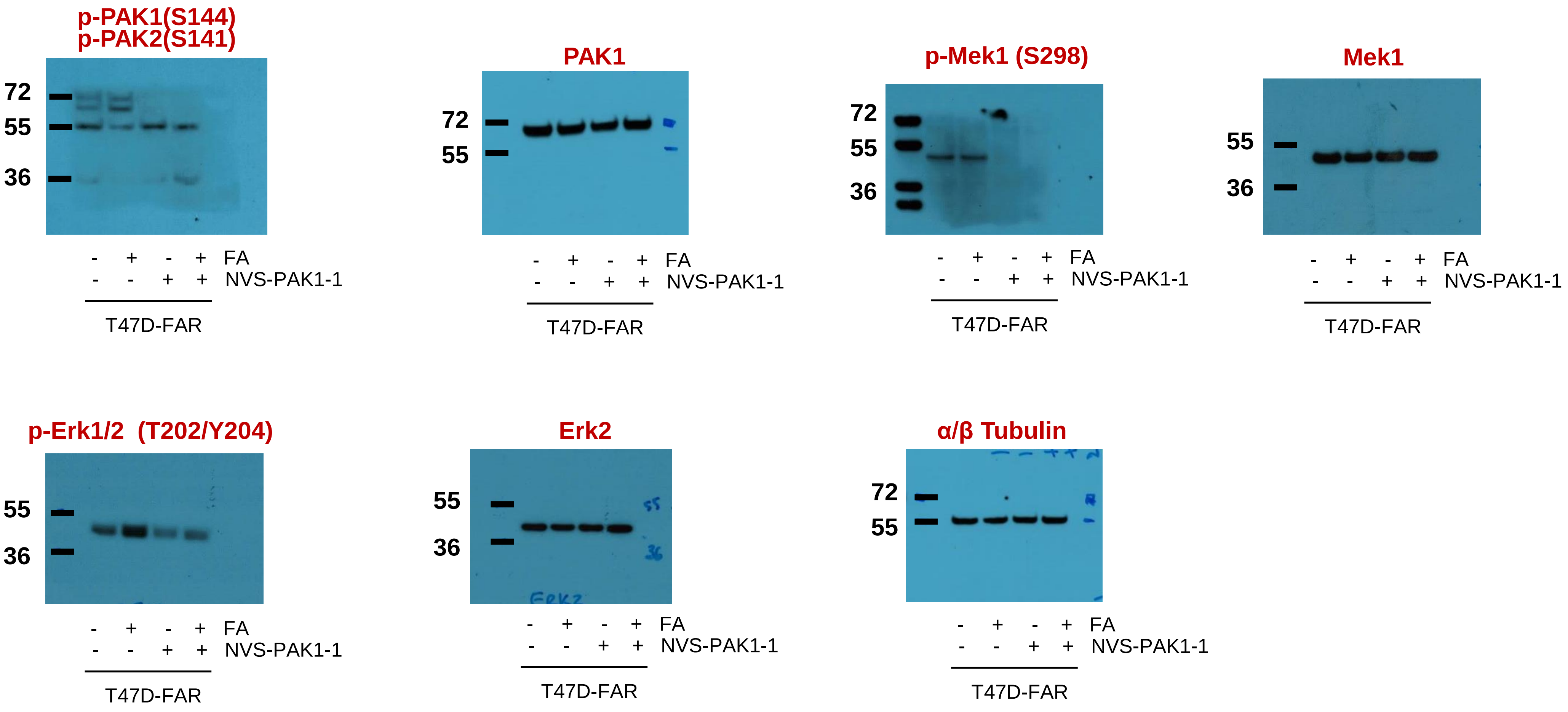

Supplementary Figure 6

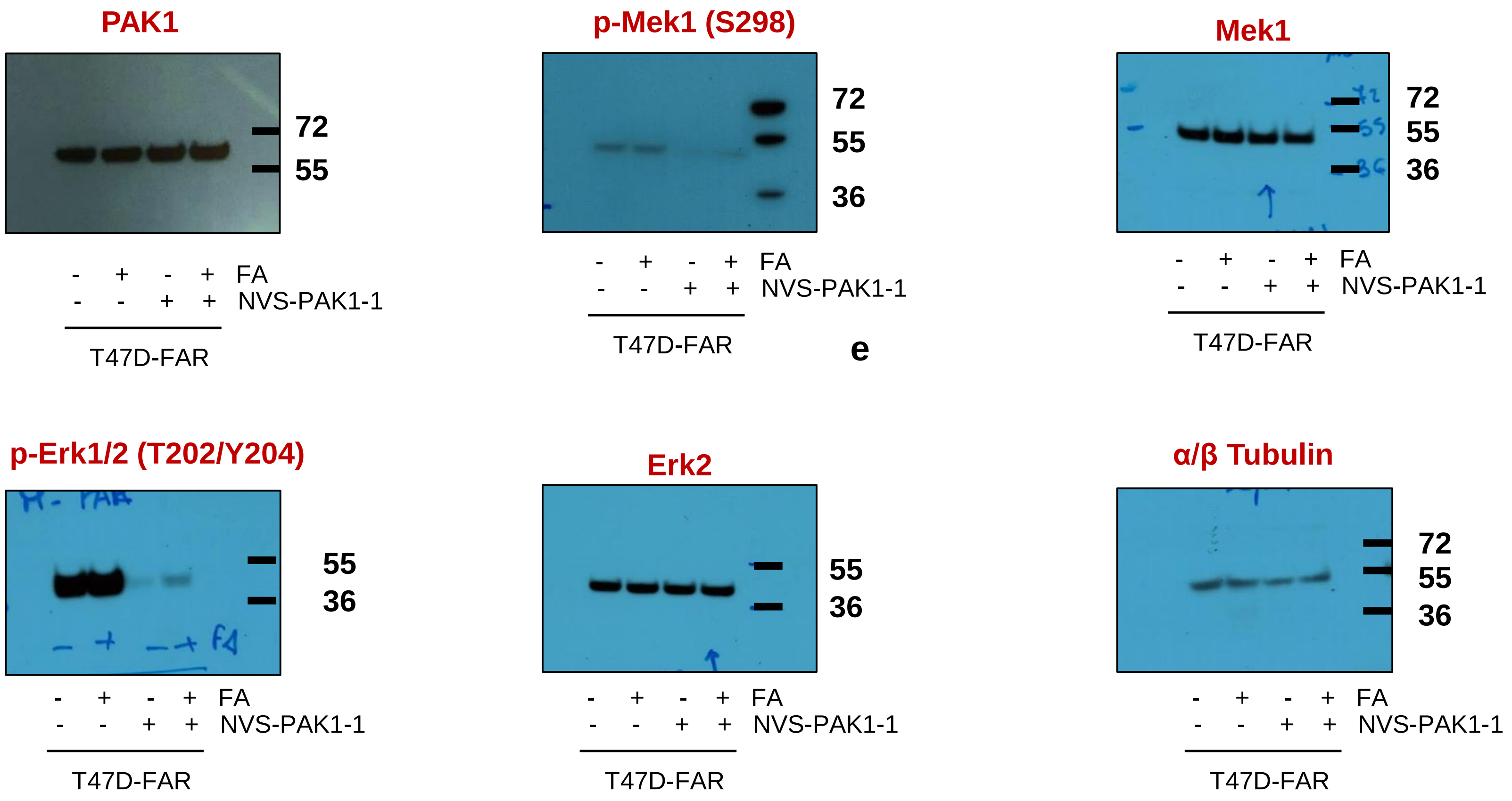

Supplement: Supplementary file 1 — Supplementary materials [file 41523_2023_556_MOESM1_ESM.pdf]
