# Supplementary material for: Pak1 pathway hyper-activation mediates resistance to endocrine therapy and CDK4/6 inhibitors in ER+ breast cancer
Source: NPJ Breast Cancer. 2023 May 31;9:48. doi: 10.1038/s41523-023-00556-9 (PMC10232479; doi:10.1038/s41523-023-00556-9)
Supplement: Supplementary file 2 — Reporting Summary [file 41523_2023_556_MOESM2_ESM.pdf]

nature portfolio

Corresponding author(s): Roberto Bianco and Luigi Formisano

Last updated by author(s): May 18, 2023

Reporting Summary

Nature Portfolio wishes to improve the reproducibility of the work that we publish. This form provides structure for consistency and transparency in reporting. For further information on Nature Portfolio policies, see our Editorial Policies and the Editorial Policy Checklist.

Statistics

For all statistical analyses, confirm that the following items are present in the figure legend, table legend, main text, or Methods section.

n/a	Confirmed	
☐	☒	The exact sample size (*n*) for each experimental group/condition, given as a discrete number and unit of measurement
☐	☒	A statement on whether measurements were taken from distinct samples or whether the same sample was measured repeatedly
☐	☒	The statistical test(s) used AND whether they are one- or two-sided<br>*Only common tests should be described solely by name; describe more complex techniques in the Methods section.*
☒	☐	A description of all covariates tested
☐	☒	A description of any assumptions or corrections, such as tests of normality and adjustment for multiple comparisons
☐	☒	A full description of the statistical parameters including central tendency (e.g. means) or other basic estimates (e.g. regression coefficient) AND variation (e.g. standard deviation) or associated estimates of uncertainty (e.g. confidence intervals)
☐	☒	For null hypothesis testing, the test statistic (e.g. *F*, *t*, *r*) with confidence intervals, effect sizes, degrees of freedom and *P* value noted<br>*Give P values as exact values whenever suitable.*
☒	☐	For Bayesian analysis, information on the choice of priors and Markov chain Monte Carlo settings
☐	☒	For hierarchical and complex designs, identification of the appropriate level for tests and full reporting of outcomes
☒	☐	Estimates of effect sizes (e.g. Cohen's *d*, Pearson's *r*), indicating how they were calculated

Our web collection on statistics for biologists contains articles on many of the points above.

Software and code

Policy information about availability of computer code

Data collection	Microsoft Excel 2016
Data analysis	We used GloMax® Analysis Software for protein quantification and proliferation assays after crystal violet staining; Biorad CFX manager 3.1 for mRNA expression ; ImageJ 1.53 software bundled with 64-bit Java 1.8.0_172 for spheroids area quantification and densitometric analysis of immunoblots; GraphPad Prism 9 and Excel 2016 for data analysis, means, standard deviation and p values calculation; CompuSyn software for synergism assays and combination index calculation. GSEA_4.2.3 software for RNAseq-based gene expression analysis.

For manuscripts utilizing custom algorithms or software that are central to the research but not yet described in published literature, software must be made available to editors and reviewers. We strongly encourage code deposition in a community repository (e.g. GitHub). See the Nature Portfolio guidelines for submitting code & software for further information.

Data

Policy information about availability of data

All manuscripts must include a data availability statement. This statement should provide the following information, where applicable:

- Accession codes, unique identifiers, or web links for publicly available datasets
- A description of any restrictions on data availability
- For clinical datasets or third party data, please ensure that the statement adheres to our policy

The original data regarding the gene expression and sequencing data (RNA-seq) are archived in GEO dataset (Accession number GSE227102).

Human research participants

Policy information about studies involving human research participants and Sex and Gender in Research.

Reporting on sex and gender	No human partecipants were included in this study
Population characteristics	No human partecipants were included in this study
Recruitment	No human partecipants were included in this study
Ethics oversight	No human partecipants were included in this study

Note that full information on the approval of the study protocol must also be provided in the manuscript.

Field-specific reporting

Please select the one below that is the best fit for your research. If you are not sure, read the appropriate sections before making your selection.

☒ Life sciences ☐ Behavioural & social sciences ☐ Ecological, evolutionary & environmental sciences

For a reference copy of the document with all sections, see nature.com/documents/nr-reporting-summary-flat.pdf

Life sciences study design

All studies must disclose on these points even when the disclosure is negative.

Sample size	Dose-response cell viability assays were conducted in quadruplicate, spheroids growth and invasion assays were conducted at least in quadruplicate, Real time-PCR were conducted in triplicate. Transwell invasion assays were conducted in triplicate. RNA-seq analysis, western blot, phalloidin staining were perfomed in triplicate. For in vivo study with tumor xenografts n= 5.
Data exclusions	No data were excluded
Replication	Data are expressed as mean at least three separate experiments performed in triplicate, unless otherwise specified.
Randomization	For in vivo sudy, mice were randomized in 4 arms of treatment when tumors reached ≥ 200mm3
Blinding	Blinding was not relevant for the preclinical study

Reporting for specific materials, systems and methods

We require information from authors about some types of materials, experimental systems and methods used in many studies. Here, indicate whether each material, system or method listed is relevant to your study. If you are not sure if a list item applies to your research, read the appropriate section before selecting a response.

Materials & experimental systems

n/a	Involved in the study	
☐	☒	Antibodies
☐	☒	Eukaryotic cell lines
☒	☐	Palaeontology and archaeology
☐	☒	Animals and other organisms
☒	☐	Clinical data
☒	☐	Dual use research of concern

Methods

n/a	Involved in the study	
☒	☐	ChIP-seq
☒	☐	Flow cytometry
☒	☐	MRI-based neuroimaging

Antibodies

Antibodies used	All antibodies used in this study are listed in Materials and Methods

Validation	All antibodies are commencially available and already validated

Eukaryotic cell lines

Policy information about cell lines and Sex and Gender in Research

Cell line source(s)	MCF-7 (ATCC® HTB-22™), T47D (ATCC® HTB-133™)
Authentication	Cell lines were authenticated by STR method
Mycoplasma contamination	All cell lines resulted mycoplasma free before use
Commonly misidentified lines (See ICLAC register)	None

Animals and other research organisms

Policy information about studies involving animals; ARRIVE guidelines recommended for reporting animal research, and Sex and Gender in Research

Laboratory animals	6-week-old female Balb/c (nu+/nu+) mice (ENVIGO)
Wild animals	N/A
Reporting on sex	Only female mice were used for xenograft growth analysis of breast cancer cell lines. Sex-based analysis was not performed because it is not relevant for breast cancer studies.
Field-collected samples	Animal weights and tumor diameters (with calipers) were measured twice weekly and tumor volume in mm3 was calculated with the formula: volume = width2 x length/2. After 6 weeks, tumors were harvested and snap-frozen in liquid nitrogen or fixed in 10% neutral buffered formalin followed by embedding in paraffin for IHC
Ethics oversight	Mouse experiments were maintained in accordance with institutional guidelines of the University of Naples Animal Care Committee and in accordance with the Declaration of Helsinki.

Note that full information on the approval of the study protocol must also be provided in the manuscript.
